# Acceptability, consideration, intention, and uptake of six common types of direct‐to‐consumer genetic tests in the Netherlands

**DOI:** 10.1002/jgc4.70142

**Published:** 2025-11-25

**Authors:** Anna Roos Leerschool, Anke Wesselius, Gowri Gopalakrishna, Maurice P. Zeegers

**Affiliations:** ^1^ CAPHRI School for Public Health and Primary Care Maastricht University Maastricht The Netherlands; ^2^ NUTRIM School of Nutrition and Translational Research in Metabolism Maastricht University Maastricht The Netherlands; ^3^ Department of Epidemiology and Data Science Amsterdam University Medical Center Amsterdam The Netherlands

**Keywords:** acceptability, attitudes, consideration, cross‐sectional survey study, decision‐making, direct‐to‐consumer genetic testing, intention

## Abstract

While direct‐to‐consumer genetic testing (DTC‐GT) has gained significant popularity, concerns persist that the public may lack adequate information and support to make well‐informed decisions and understand test results. Several types of DTC‐GT are on the market, each with distinct purposes and risks. The expected user population may differ per type of DTC‐GT, suggesting a need for tailored information materials. Considering six different types of DTC‐GT, this paper aims to identify how people's acceptability of DTC‐GT and their interest in undergoing a DTC‐GT within the next year (intention) and in the future (consideration) may differ depending on individuals' characteristics or the type of DTC‐GT. An online cross‐sectional survey was conducted in April 2022 among Dutch adults. Generalized linear models determined factors associated with DTC‐GT acceptability, consideration, and intention. Open‐ended responses were analyzed using inductive content analysis in MaxQDA. Of 907 respondents, 34 (3.7%) had purchased a DTC‐GT, with the majority opting for ancestry tests. Health‐related tests had the highest consideration and intention but were deemed the least acceptable to undergo without a healthcare professional. Open‐ended responses supported quantitative findings on the differences in acceptability, consideration, and intention across test types. Overall, few respondents intended to undergo a test within the next year. Factors influencing DTC‐GT acceptability, consideration, and intention overlapped by the test type. The most common factors, age and education level, were both inversely associated with the outcomes. This study suggests that the Dutch public is mostly interested in health‐related DTC‐GT but does not find them acceptable without professional support. Ensuring that DTC‐GT information is comprehensible for younger and less educated individuals is crucial. Genetic counselors could provide valuable expertise in developing these materials.

**What is known about this topic?**

The Dutch public's interest in and uptake of different types of DTC‐GT currently on the market remains largely unexplored.

**What this paper adds to the topic?**

This paper reveals that the Dutch public is mainly interested in health‐related DTC‐GT but prefers professional support when undergoing these tests. It highlights the need for tailored information materials to accommodate younger individuals and those with less education.

## 1 INTRODUCTION

Over the past decade, direct‐to‐consumer genetic testing (DTC‐GT) without the involvement of healthcare professionals has gained significant popularity (Khan & Mittelman, 2018). Since 2016, the global DTC‐GT market has expanded exponentially, surpassing 41 million genotyped consumers worldwide by March 2024 (Janzen, 2024). Increased publicity, decreasing costs, and growing public interest in proactive health management likely contribute to this rise in DTC‐GT uptake (Cherkas et al., 2010; Khan & Mittelman, 2018). By April 2020, over 50 DTC‐GT providers, both domestic and international, were accessible to Dutch consumers online. Current DTC‐GT options include, but are not limited to, health‐related tests (e.g., disease‐risk, carrier, diet and metabolism, and pharmacogenetic tests), ancestry tests, sport‐related tests, and tests for entertainment.

Despite the purported benefits of DTC‐GT, such as increased autonomy, increased social engagement in genetics, identifying individuals who might otherwise not have known they were at risk, and encouraging positive lifestyle changes based on genetic insights (Blout Zawatsky et al., 2023; Chung & Ng, 2016; Rigter et al., 2020; Vayena, 2015), there are significant concerns about the adequacy of information and support available to consumers. For example, a recent content analysis of health‐related DTC‐GT sellers' websites accessible to Dutch consumers found that the information provided is often suboptimal (Bruins et al., 2024). Without adequate pre‐ and post‐test information and support, many may struggle to make well‐informed decisions or fully comprehend the implications of their test results (Cernat et al., 2022; Pavarini et al., 2021; Stewart et al., 2018). Misunderstanding DTC‐GT results may cause psychological distress, poor health choices, and avoidable consultations with healthcare professionals (Cernat et al., 2022; Rafiq et al., 2015). Moreover, each type of DTC‐GT carries unique challenges alongside common concerns about privacy, data quality (including high false positive rates, particularly for rare pathogenic variants), and the reliability of third‐party interpretation services (including the misclassification of benign variants as pathogenic) (Moscarello et al., 2019; Tandy‐Connor et al., 2018; Weedon et al., 2021). Ancestry tests, for example, can reveal unexpected relationships or new family members (Crawshaw, 2018; Larmuseau, 2019), while complex diet and metabolism results need to be communicated so that people can make appropriate changes to dietary behaviors (Blout Zawatsky et al., 2023).

Given these complexities, comprehensive pre‐ and post‐test materials are needed to help consumers navigate the DTC‐GT options available and, when needed, appropriately act on the results. Genetic counselors are uniquely positioned to provide valuable expertise in developing these materials (Blout Zawatsky et al., 2023; Hudson et al., 2007; National Society of Genetic Counselors, 2019). In the Netherlands, genetic counseling services are an integral part of clinical genetic care, primarily available through university medical centers. According to a 2019 publication, the number of genetic counselors in the Netherlands was estimated at 55 (Abacan et al., 2019). Moreover, as of December 1st 2024, there are 208 clinical geneticists (BIG‐register, 2024), with the current workforce reportedly sufficient to meet the demand for clinical genetic services (Capiciteitsorgaan, 2022). However, access to these services typically requires a referral from a general practitioner, and they are focused on clinical, rather than direct‐to‐consumer, testing contexts.

Understanding the characteristics of the expected DTC‐GT user population can help tailor public information materials effectively. Our research team recently conducted a cross‐sectional survey of the Dutch population's acceptability, consideration, intention, and uptake of six different types of DTC‐GT. Previously published work based on this survey focused on the survey findings for disease‐related DTC‐GT (Leerschool et al., 2024). However, the characteristics associated with other types of DTC‐GT remain understudied. The expected user populations may differ per type of DTC‐GT, suggesting the need for tailored information materials specific to each test type.

This paper aims to identify whether specific characteristics influence the acceptability, consideration, and intention to engage in DTC‐GT and to evaluate whether these factors differ across six different DTC‐GT types. The current manuscript is based on the same cross‐sectional survey described previously (Leerschool et al., 2024). Previous results from our paper on disease‐related DTC‐GT in the Netherlands showed low acceptability without professional support, intention to test within a year, and uptake. However, 30% of respondents considered undergoing a disease‐related test in the distant future (Leerschool et al., 2024). Similar trends have been observed in other studies (Bos et al., 2022), yet the Dutch public's interest in other types of DTC‐GT remains largely unexplored.

## 2 METHODS

### 2.1 Design

This cross‐sectional analysis assesses the acceptability, consideration, intention, and uptake of six different types of DTC‐GT in the Dutch population. It is based on the questionnaire constructed in 2017 by Stewart et al. (2018) on disease‐related DTC‐GT and described previously (Leerschool et al., 2024). The six categories of DTC‐GT included in this study were selected based on previous reports of health‐related DTC‐GT available to consumers in the Netherlands (Rigter et al., 2020) and the global popularity of certain test types. They reflect distinct consumer motivations and test purposes, as well as industry and marketing practices. A detailed definition of each category, along with examples, can be found in the Appendix S2. Briefly, these categories include disease‐related tests (e.g., genetic disease‐risk tests and carrier screening tests), sport‐related tests, pharmacogenetic tests, diet and metabolism tests, ancestry tests, and entertainment tests focusing on fun facts. The questionnaire underwent pretesting conducted by the internet research agency *Flycatcher* (Maastricht, The Netherlands) using a small sample of participants who shared characteristics with the target population (Flycatcher Internet Research, 2024). Participation in the survey was voluntary, and all responses were treated as confidential and anonymous, in accordance with the General Data Protection Regulation (GDPR). The survey was administered in Dutch in April 2022 and received approval from the Ethics Review Committee of Maastricht University (FHML‐REC/2021/085). This study's reporting conforms to the Strengthening the Reporting of Observational Studies in Epidemiology (STROBE) guidelines (von Elm et al., 2008) (Appendix S1).

### 2.2 Data collection and participants

Full details on the participants and data collection are described elsewhere (Leerschool et al., 2024). In brief, a sample representative of the Dutch adult population (18+) was drawn from Flycatcher's ISO‐certified online panel, stratified by age, gender, education, and province. Eligible participants received a personalized email containing a link to the self‐administered online questionnaire, which included an authentication question, informed consent, and participant information. Participants could ask questions via email before or during the survey. The survey opened with an introduction to the different types of DTC‐GT and included links to educational videos and webpages (Appendix S2). Following this, participants completed a 16‐question survey with multiple‐choice, yes/no, and open‐ended questions on DTC genetic testing related to disease, sport, diet and metabolism, ancestry, pharmacogenetics, and entertainment. It took approximately 15 min to complete. To improve the response rate, a reminder email was sent after 1 week. Participants could return to the survey at any point during the study period to continue where they left off or to change their answers before submitting the questionnaire. Non‐respondents included those who did not access the survey via the emailed link, declined to provide consent, left the survey incomplete, or, based on Flycatcher's quality control criteria, did not complete the survey seriously.

In addition to the previously described survey content (Leerschool et al., 2024), respondents who answered “yes” to whether they had ever purchased a DTC‐GT were asked open‐ended questions about the brand of test they had purchased and their opinion about the test. The outcome parameters acceptability, consideration, and intention, are described in Leerschool et al. (2024), and were all rated on a 5‐point Likert scale. Acceptability was defined as the degree to which respondents found it acceptable for private companies to provide DTC‐GT results directly to consumers, without the mandatory involvement of a professional. Consideration assessed the interest in future DTC‐GT uptake, while intention gauged the interest in undergoing DTC‐GT within a year. Additionally, respondents were offered the opportunity to provide a qualitative explanation for their answers to the questions on acceptability, consideration, and intention. This was asked as “Can you briefly explain your answers to question (question number)?” There was no character limit for open‐ended responses. Prior awareness of DTC‐GT, and independent variables (including demographic factors and health‐related characteristics) remain unchanged from those previously reported (Leerschool et al., 2024). Prior awareness of DTC‐GT was determined by asking the following two questions: “Prior to participating in this research, had you ever heard of DTC‐GT?” (yes/no answer). If yes, the question “Of which type of DTC‐GT had you heard before?” was asked. Multiple answer options were accepted. Participants were asked for consent before responding to a question regarding religion (yes/no), in compliance with the GDPR.

### 2.3 Data analysis

The analyses conducted in this study were primarily exploratory, aimed at understanding public perceptions and attitudes toward DTC‐GT across a range of independent and dependent variables. There was no predefined primary outcome, as the goal was to identify key patterns of interest, particularly variations in acceptability and interest in different types of DTC‐GT. These exploratory analyses provide a foundation for future research, which may involve more focused and hypothesis‐driven investigations.

#### 2.3.1 Quantitative analysis

To determine factors associated with the acceptability, consideration, and intention of DTC‐GT, generalized linear models with a multinomial distribution and a cumulative logit link function were used. Variables with a significance level of *p* < 0.20 in univariable analyses were selected for inclusion in the multivariable models. The linearity of the continuous variables age and self‐rated health, with each outcome, was assessed using the Box‐Tidwell transformation. A *p*‐value threshold of <0.01 on the test of parallel lines was used to determine any statistically significant violations of the proportional odds assumption in the multivariable models. Friedman tests were performed to examine the differences in acceptability, consideration, and intention between the different types of DTC‐GTs. Higher mean ranks indicate more favorable ratings. Pairwise Wilcoxon signed‐rank tests between each pair of related samples were conducted with Bonferroni correction to control for multiple comparisons. All analyses were carried out using SPSS Version 28.0.1.0(142) and Stata Release 19, with a significance level set at *p* < 0.05.

#### 2.3.2 Qualitative analysis

Responses to open‐ended questions were analyzed using inductive content analysis in MaxQDA (Rädiker & Kuckartz, 2020). ARL reviewed and coded all responses with guidance from GG, an experienced qualitative researcher. To ensure quality, category definitions were added to codes, detailing when each should be used and how it distinguishes from others (Kuckartz, 2014; Schreier, 2012). Additionally, specific coding rules were established: (1) each answer, being only a few lines long, was coded in its entirety, (2) choosing the whole answer as the coding unit, also meant that each code could only be assigned to a response once, preventing double coding of repeated information, (3) when respondents referred to a previous answer, “wrongly placed” answers were coded for the correct question, (4) unusable answers, such as “none” or “no” were assigned to an “unusable” category. Codes were condensed into themes. AW checked 10% of codes and agreement was reached through discussion.

## 3 RESULTS

Of the 1964 invited panel members, 1265 (64%) responded to the questionnaire. Among these, 10 were excluded due to low‐quality responses (fully completed questionnaires that failed to meet Flycatcher's quality control standards), and 348 either withheld consent, withdrew or provided incomplete data, leaving 907 respondents for analysis. A full description of the study sample is provided in Leerschool et al., (2024). Comparisons between respondents and non‐respondents indicated no significant differences in age or gender; however, non‐respondents were more likely to have lower educational attainment and less likely to have higher education (*χ* ^2^ (2, *N* = 1971) = 6.65, *p* = 0.036).

A total of 38.9% of respondents had prior awareness of DTC‐GT. Awareness was greatest for ancestry testing (32.0%), followed by disease‐related (19.7%), entertainment (6.2%), diet and metabolism (5.8%), pharmacogenetics (5.2%) and sport tests (2.6%).

### 3.1 Uptake of DTC‐GT

**FIGURE 1:**
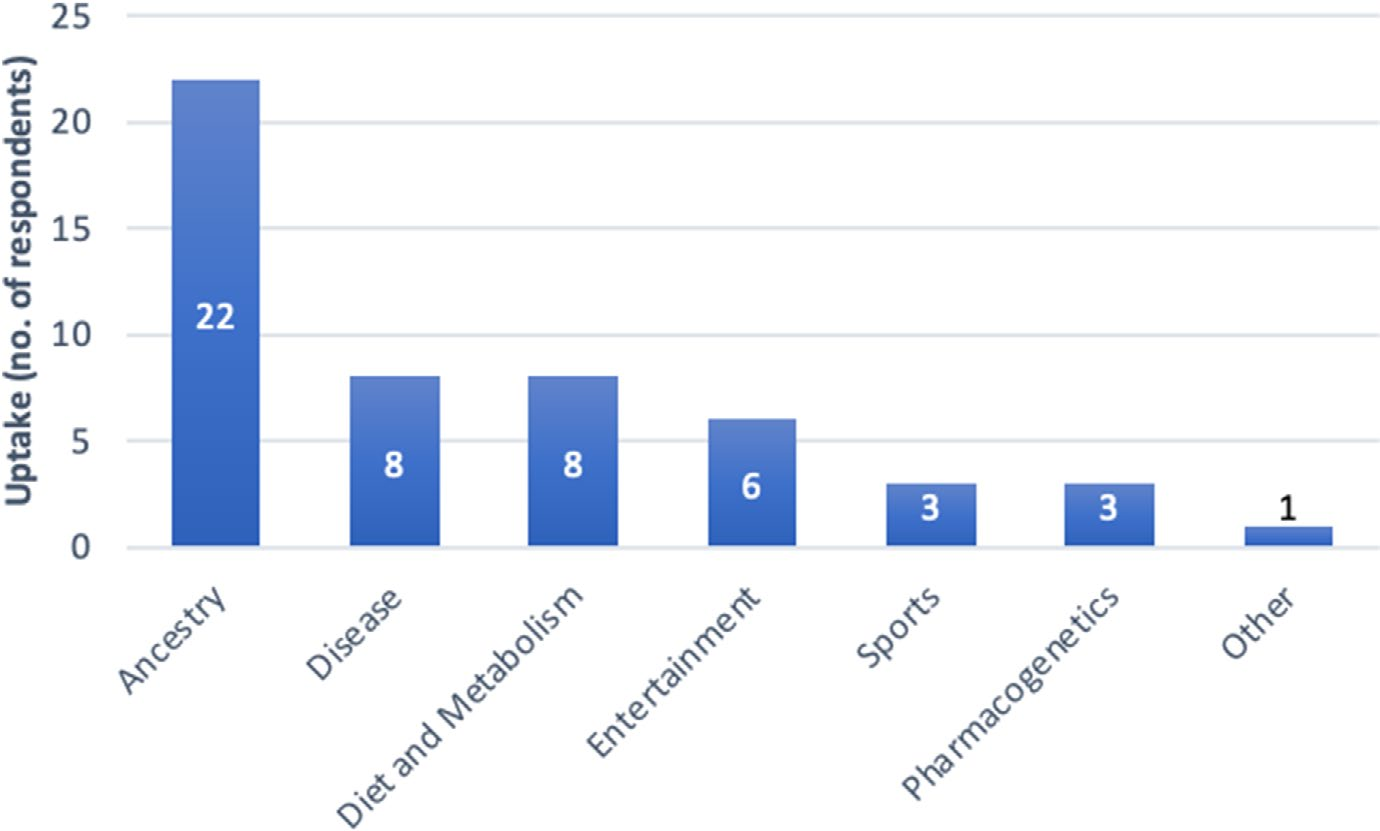
Reasons for DTC‐GT uptake (*n* = 34).

Thirty‐four of 907 respondents (3.7%) had previously purchased a DTC‐GT. Of these, the majority purchased a DTC‐GT for ancestry purposes (*n* = 22, 64.7%), followed by disease‐related purposes (*n* = 8, 23.5%), diet and metabolism (*n* = 8, 23.5%), entertainment (*n* = 6, 17.6%), pharmacogenetics (*n* = 3, 8.8%), sport (*n* = 3, 8.8%), and other purposes (*n* = 1, 2.9%) (Figure 1). The respondent who selected the “other” answer option, underwent paternity testing.

Eight respondents shared the brand of test they had purchased: MyHeritage (*n* = 3), iGene (*n* = 2), 23 and Me (*n* = 1), Ancestry DNA (*n* = 1), Circle DNA (*n* = 1). Thirty‐one of 34 respondents who had purchased a DTC‐GT shared their impressions of the test. Impressions focused on: (1) practical matters such as high costs and the ease of the sampling and shipping process, (2) privacy concerns, (3) general impressions such as “exciting”, “ok” and “clear”, and (4) DTC‐GT results. Impressions about the DTC‐GT results included positive impressions (interesting, fun, plausible and useful results), negative impressions (vague, confronting, or unremarkable results), and neutral impressions (surprising results).

### 3.2 Acceptability, consideration, and intention of DTC‐GT

**FIGURE 2:**
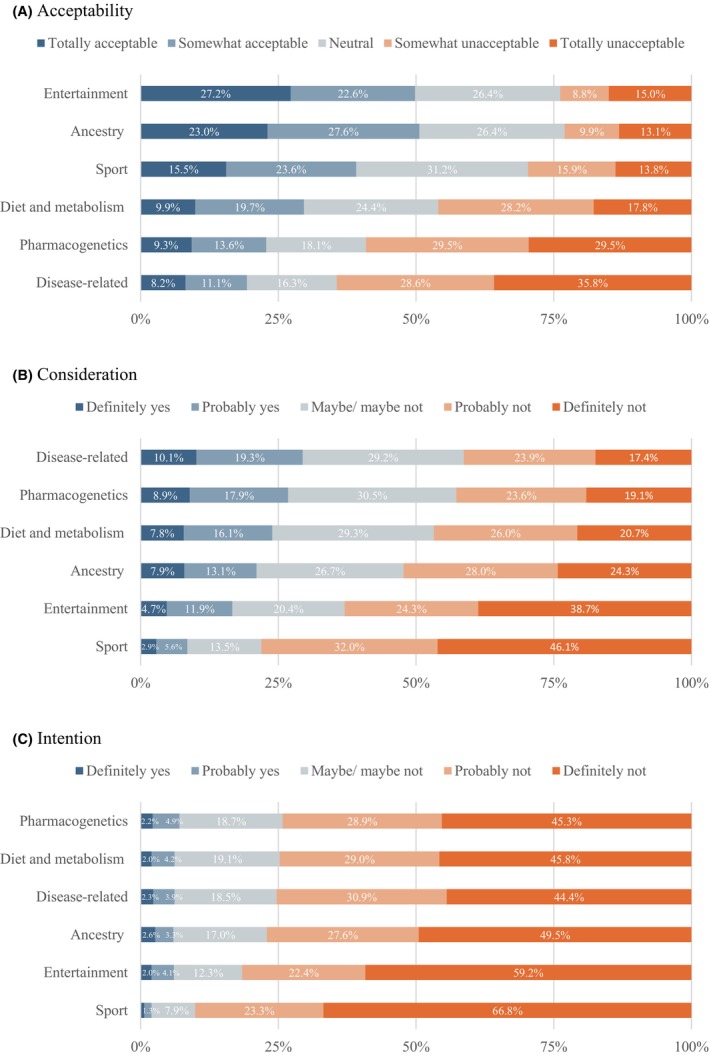
(A) Acceptability, (B) consideration, and (C) intention of six common types of DTC‐GT. Having biological children and being religious showed no associations with any outcome for any of the DTC‐GT types.

Figure 2 shows the acceptability, consideration, and intention of six common types of DTC‐GT (Table S1). Friedman tests showed statistically significant differences in acceptability, consideration, and intention across the six types of DTC‐GT (Table S2).

#### 3.2.1 Acceptability

DTC‐GT for ancestry (mean rank = 4.21) and entertainment (mean rank = 4.21) purposes were rated as the most acceptable tests to undergo without the support of a healthcare professional. A total of 50.6% and 49.8% of respondents found ancestry and entertainment tests, respectively, to be totally or somewhat acceptable. Pairwise comparison indicated no statistically significant difference in acceptability between these two test types (*p* > 0.999). Following ancestry and entertainment tests, sport‐related tests were considered acceptable by 39.1% of respondents, with a mean rank of 3.84. Health‐related tests were rated least acceptable: diet and metabolism (29.7%; mean rank = 3.32), pharmacogenetic (22.8%; mean rank = 2.85) and disease‐related tests (19.3%; mean rank = 2.57).

#### 3.2.2 Consideration

Respondents most considered undergoing disease‐related (29.4%; mean rank = 4.10) and pharmacogenetic tests (26.8%; mean rank = 3.99) at some point in the future. This was followed by diet and metabolism (23.9%; mean rank = 3.82), ancestry (21.1%; mean rank = 3.61), entertainment (16.6%; mean rank = 3.01) and sport tests (8.5%; mean rank = 2.47). Pairwise comparisons showed no statistically significant difference in consideration between disease‐related and pharmacogenetic tests (*p* > 0.999), pharmacogenetic and diet and metabolism tests (*p* = 0.670), and diet and metabolism and ancestry tests (*p* = 0.304).

#### 3.2.3 Intention

Most respondents did not intend to undergo a test within the next year. Responses for definitely or probably yes ranged from 2.0% for sport tests to 7.1% for pharmacogenetic tests. Similarly to consideration, intention was lowest for sport tests (mean rank = 2.89), followed by entertainment tests (mean rank = 3.28). Pairwise comparisons showed no statistically significant differences in intention between ancestry (mean rank = 3.59), diet and metabolism (mean rank = 3.74), disease‐related (mean rank = 3.75), and pharmacogenetic (mean rank = 3.77) tests.

### 3.3 Uni‐ and multivariable analyses

**FIGURE 3:**
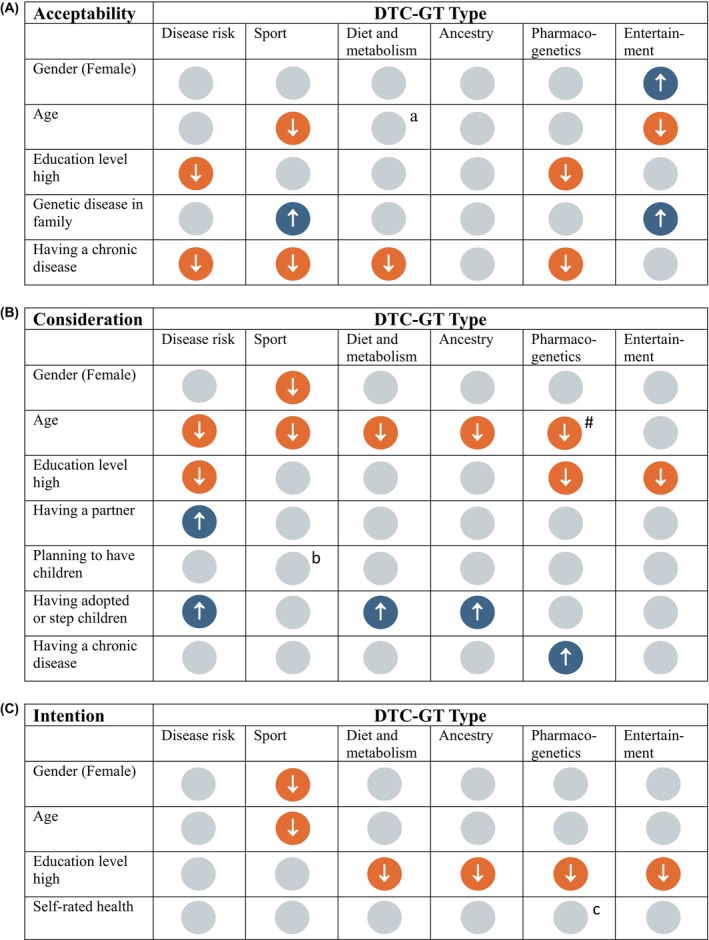
Multivariable results for the association between independent variables and (A) acceptability, (B) consideration, and (C) intention, of six types of DTC‐GT. Legend: Blue = positive association, orange = negative association, and gray = no association; # Older age increased the likelihood of “definitely not” considering pharmacogenetic testing (*b* = −0.022, *p* < 0.001, partial proportional odds model). Without religion in the model: (a) *b* = −0.452, *p* = 0.041, (b) *b* = 0.438, *p* = 0.047, and (c) *b* = −0.169, *p* = 0.050.

The linearity assessment between age (in years) and the acceptability of all DTC‐GT types, except pharmacogenetic testing, indicated a violation of linearity. Therefore, for these analyses, age was treated as a categorical variable in both uni‐ and multivariable analyses. The assumption of proportional odds was violated for the multivariable model of pharmacogenetic test consideration. The model was re‐estimated using a partial proportional odds model in Stata using the **‘**autofit’ option (Williams, 2006). Only the variable age in years violated the proportional odds assumption (*p* < 0.001). For this variable, results are reported per outcome threshold. Positive coefficients indicate that higher values of the explanatory variable (age) increase the probability that a respondent is in a higher outcome category than the current one. Negative coefficients indicate that older age increases the probability of being in the current or a lower category (Williams, 2006) (Table S7). Twenty‐eight respondents (1.6%) declined consent to answer a question on religion. To assess the impact of this partially missing variable, multivariable models were conducted both including and excluding religion, where appropriate. Results from multivariable models including and excluding religion are presented below to ensure transparency (Figure 3, Tables [Link], [Link]).

#### 3.3.1 Acceptability

##### Disease‐related tests

More highly educated respondents (*b* = −0.549, *p* = 0.001) and those with a chronic disease (*b* = −0.337, *p* = 0.010), were less likely to find disease‐related tests acceptable without the involvement of a healthcare professional.

##### Sport tests

Older age (60+) (*b* = −0.574, *p* = 0.011) and having a chronic condition (*b* = −0.325, *p* = 0.031) were associated with lower acceptability of sport tests, while a family history of genetic disease was associated with increased acceptability (*b* = 0.353, *p* = 0.024).

##### Diet and metabolism tests

Acceptability of diet and metabolism DTC‐GT was lower among respondents with a chronic disease (*b* = −0.304, *p* = 0.034). Without religion in the model, older age was also statistically significantly associated with lower acceptability of diet and metabolism tests (*b* = −0.452, *p* = 0.041).

##### Ancestry tests

No factors were associated with the acceptability of ancestry tests.

##### Pharmacogenetic tests

More highly educated respondents (*b* = −0.420, *p* = 0.011) and those with a chronic disease (*b* = −0.403, *p* = 0.002) were less likely to find pharmacogenetic tests acceptable.

##### Entertainment tests

Older age (60+) was associated with lower acceptability of entertainment tests (*b* = −0.716, *p* = 0.002), while female respondents (*b* = 0.307, *p* = 0.018) and those with a family history of genetic disease (*b* = 0.322, *p* = 0.041) were more likely to find entertainment tests acceptable.

#### 3.3.2 Consideration

##### Disease‐related tests

Older age (per 1 year increase *b* = −0.025, *p* < 0.001) and higher education (*b* = −0.417, *p* = 0.017) were associated with lower consideration of disease‐related tests. On the other hand, there was increased consideration among respondents with a partner (*b* = 0.307, *p* = 0.037) and adopted children/ stepchildren (*b* = 0.486, *p* = 0.013).

##### Sport tests

Females (*b* = −0.416, *p* = 0.002) and older respondents (per 1 year increase *b* = −0.017, *p* = 0.002) were less likely to consider sport tests. Without religion in the model, respondents planning to have children were more likely to consider sport tests (*b* = 0.438, *p* = 0.047).

##### Diet and metabolism tests

Older age was associated with lower consideration of diet and metabolism tests (per 1 year increase *b* = −0.018, *p* < 0.001), while having adopted children/ stepchildren was associated with increased consideration (*b* = 0.461, *p* = 0.017).

##### Ancestry tests

Older age was associated with lower consideration of ancestry tests (per 1 year increase *b* = −0.013, *p* = 0.010), while having adopted children/ stepchildren was associated with increased consideration (*b* = 0.419, *p* = 0.030).

##### Pharmacogenetic tests

Higher education decreased consideration of pharmacogenetic tests (*b* = −0.405, *p* = 0.017), while people with a chronic disease were more likely to consider pharmacogenetic tests than those without a chronic disease (*b* = 0.257, *p* = 0.048). Older age increased the likelihood of “definitely not” considering pharmacogenetic testing (*b* = −0.022, *p* < 0.001, partial proportional odds model).

##### Entertainment tests

Higher education decreased consideration of entertainment tests (*b* = −0.424, *p* = 0.017).

#### 3.3.3 Intention

##### Disease‐related tests

No factors were associated with the intention to undergo disease‐related tests.

##### Sport tests

Females (*b* = −0.418, *p* = 0.005) and older respondents (per 1 year increase *b* = −0.014, *p* = 0.026) showed less intention to undergo sport tests.

##### Diet and metabolism, ancestry, pharmacogenetic and entertainment tests

Higher education decreased intention to undergo diet and metabolism (*b* = −0.434, *p* = 0.014), ancestry (*b* = −0.426, *p* = 0.016), pharmacogenetic (*b* = −0.646, *p* < 0.001), and entertainment (*b* = −0.497, *p* = 0.005) tests. Without religion in the model, better self‐rated health was associated with lower intention to take a pharmacogenetic DTC‐GT (per 1 point increase *b* = −0.169, *p* = 0.050).

### 3.4 Qualitative results

**FIGURE 4:**
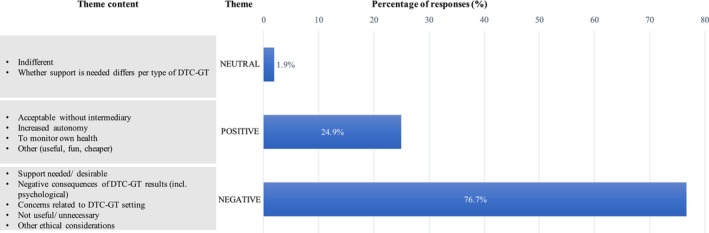
Reasons influencing perceived acceptability of DTC‐GT without a professional. The percentage of responses that mention each theme is based on the available responses for this question (*n* = 519). Percentages do not add up to 100% as some responses mention more than one theme.

**FIGURE 5:**
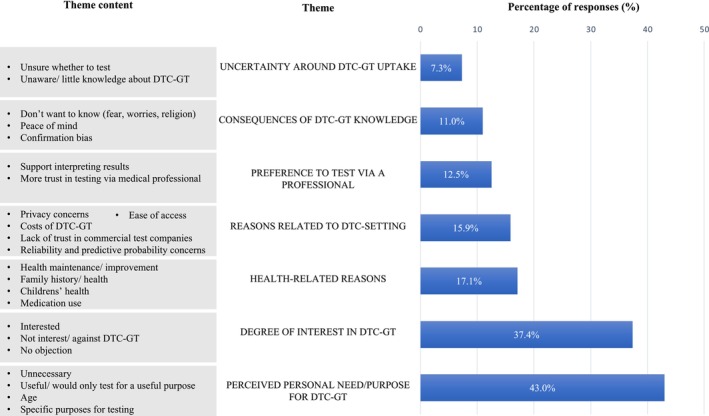
Reasons influencing DTC‐GT consideration decisions. The percentage of responses that mention each theme is based on the available responses for this question (*n* = 672). Percentages do not add up to 100% as some responses mention more than one theme.

**FIGURE 6:**
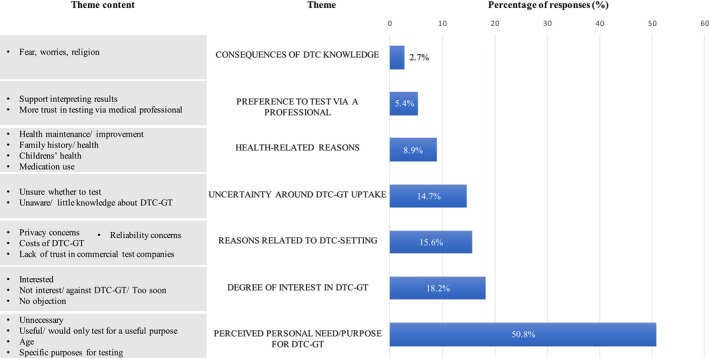
Reasons influencing DTC‐GT intention decisions. The percentage of responses that mention each theme is based on the available responses for this question (*n* = 518). Percentages do not add up to 100% as some responses mention more than one theme.

The themes (in italics) identified from the qualitative analysis of open‐ended responses to the questions on acceptability, consideration, and intention are illustrated in Figures 4, 5, 6 and in the following text.

#### 3.4.1 Acceptability

Three major themes were identified from the responses to the open‐ended question on acceptability (*n* = 519). These were *negative*, *positive*, and *neutral perceptions* about DTC‐GT companies providing test results directly to the consumer, without the involvement of a professional (Figure 4).

##### Negative perceptions

Of the 519 respondents who completed the open‐ended question about acceptability, most shared *negative perceptions* (76.5%). The primary concern was the need for a professional for post‐test support, particularly for health‐related tests and adverse results. Respondents felt they lacked sufficient knowledge about DTC‐GT and desired guidance interpreting results to prevent wrong conclusions and health risks. Some expressed that not everyone should have access to genetic information, calling it irresponsible yet inevitable. They suggested collaboration between companies and medical professionals for pre‐ and post‐test support and emphasized the importance of independent control and consumer protection for DTC‐GT companies.

Additional concerns included the risks of psychological impacts such as unnecessary worry and stress, doubts about the reliability and privacy of DTC results, and a general distrust of commercial DTC‐GT companies. Ethical considerations mentioned were the possible burden on the healthcare system, the violation of family members' right not to know, and possible discrimination based on health status. Some respondents did not find DTC‐GT useful or necessary unless there was a specific medical indication.

##### Positive perceptions

Positive perceptions (24.5%) centered around the topics of testing being acceptable without a professional, autonomy, monitoring one's own health, and “other” perceptions.

Non‐health‐related tests were generally considered acceptable without professional involvement, with results deemed easier to interpret and harmless to share directly with consumers. Autonomy was emphasized, with respondents viewing the choice to undergo and disclose DTC‐GT results as a personal responsibility, affirming the right to information about one's own body and the preference for privacy.

Respondents also expressed a desire for health‐related DTC‐GT to proactively manage health concerns, especially when traditional medical channels were inaccessible. Additional positive perceptions included the utility of DTC‐GT (e.g., for research), its entertainment aspect, and its cost‐effectiveness compared to conventional genetic testing.

##### Neutral perceptions

Neutral perceptions (1.9%) included being indifferent to whether DTC‐GT companies share results directly with the consumer, and whether support is needed depends on the type of DTC‐GT.

#### 3.4.2 Consideration and intention

Figures 5 and 6 illustrate reasons for considering and intending to use DTC‐GT, identifying seven main themes: (1) *perceived personal need/ purpose for testing*, (2) *degree of interest in DTC‐GT*, (3) *reasons related to DTC setting*, (4) *uncertainty around DTC‐GT uptake*, (5) *health‐related reasons*, (6) *preference to test* via *a professional*, and (7) *consequences of DTC‐GT knowledge*.

Among the respondents who answered the open‐ended questions on consideration (*n* = 672) and intention (*n* = 518), the primary reasons for considering and intending to use DTC‐GT were their *perceived personal need/ purpose for testing* and their *degree of interest*. Curiosity, fun, and the desire for knowledge were common motivators, while others cited no interest or opposition to testing. Many respondents found testing within a year premature due to a lack of readiness or necessity or a preference to wait for cheaper, improved tests. Many viewed DTC‐GT as unnecessary, considering it only for specific purposes or medical necessity. Others were interested in DTC‐GT for non‐vital reasons, preferring to leave the more “serious” tests in the hands of a medical professional. Older respondents generally felt less need to test, although there was also mention of considering DTC‐GT when older i.e., when taking medication (pharmacogenetic testing), or when having a higher disease risk. A few specific purposes for DTC‐GT consideration were mentioned such as: relationship testing, testing to help others, for family reasons, for research purposes, or to seek clarity or confirmation.

The *perceived need/ purpose for testing* is closely linked to the theme *health‐related reasons*, as respondents considered/ intended to test for health maintenance and improvement, or their current or future children's health, while feeling healthy was a reason not to test. Respondents considered/ intended to test for current conditions, symptoms, medication use, or as a health screening tool for early action and personalized prevention. A family history of certain diseases or medication side effects was mentioned as another reason to test, to assess one's risk of developing similar conditions or adverse effects.

*Preference to test* via *a (medical) professional*, was linked to trust, accurate interpretation of results, and preventing wrong conclusions. Related to requiring a purpose to test, respondents mentioned testing when medically indicated or recommended by a doctor, which could also save personal costs, acknowledging DTC‐GT as an alternative when doctors are unwilling to test. Medical tests were deemed more appropriate with professional support, while DTC‐GT was accepted for entertainment and ancestry purposes. Making pharmacogenetic tests available through GPs was also suggested.

Considerations and intentions related to the *DTC setting* included costs, privacy concerns, limited reliability and predictive probability of results, and distrust in commercial companies. Respondents valued the ease of access of DTC‐GT, which was seen as increasing autonomy and allowing testing without burdening the healthcare system or involving health insurance.

Furthermore, perceived *consequences of DTC‐GT results* influenced decisions. Some avoided testing due to anticipated stress, fear, or religious reasons, while others saw it as providing security and peace of mind. Concerns about confirmation bias were also noted.

Uncertainty around DTC‐GT uptake stemmed from unfamiliarity and lack of knowledge, with respondents wanting more information before deciding to test.

## 4 DISCUSSION

### 4.1 Acceptability, consideration, intention, and uptake of different types of DTC‐GT

The findings from this study suggest specific patterns in the Dutch public's acceptance, consideration, and intention regarding DTC‐GT. While health‐related tests are viewed with concern and are considered less acceptable without professional involvement, there is significant interest in these tests for future use. This trend suggests that although people are cautious, there is an underlying curiosity or perceived utility that encourages consideration, even if immediate uptake is limited. This appears to be particularly the case for disease‐related and pharmacogenetic tests, where acceptability was lower than consideration. This suggests a complex relationship between the perceived value of these tests and their acceptability without the mandatory support of a healthcare professional. Interestingly, our data imply that while people may appreciate the potential benefits of such tests, barriers such as distrust in DTC‐GT companies or lack of support temper their enthusiasm. Qualitative responses shed light on the possible reasons behind the lower acceptability of health‐related DTC‐GT, including, most notably, the desire for support with interpreting results to prevent health risks. A similar appeal for expert assistance has been observed in many earlier studies (Pavarini et al., 2021; Ruhl et al., 2019; Vermeulen et al., 2014). The preference to test via a (medical) professional aligns with studies showing that people trust doctors more than DTC‐GT companies (Bayer et al., 2021; Critchley et al., 2015; Metcalfe et al., 2018), a sentiment shared by the respondents in this study. Qualitative responses also highlighted fears of psychological consequences from health‐related DTC‐GT, consistent with prior research (Gollust et al., 2012), including a review of six European studies indicating concerns about results causing distress and anxiety as primary reasons for abstaining from DTC‐GT (Hoxhaj et al., 2020).

The hesitation to undergo health tests without support may play a role in why the uptake of ancestry DTC‐GT is higher than health‐related tests, reflecting global trends in the popularity of ancestry testing (Janzen, 2024; Metcalfe et al., 2018; Ruhl et al., 2019). Greater acceptability of non‐health‐related tests may be due to their perceived lower risk and easier result interpretation, possibly leading to fewer consequences. Similar findings were reported among genetic counselors, with 97.1% finding ancestry testing acceptable in a DTC‐GT setting, followed by paternity testing (57.6%), trait testing (46.0%), carrier screening (23.6%), pharmacogenomics (20.2%), adult‐onset conditions (6.7%) and cancer (6.0%) (Hsieh et al., 2021).

Despite some variations across test types, both uptake and intention to undergo DTC‐GT were low, in agreement with previous research on (health‐related) DTC‐GT uptake in the Netherlands (Bos et al., 2022). In the current study, many respondents felt undergoing DTC‐GT within a year was premature, citing a lack of readiness or necessity, or a preference to wait for cheaper and improved tests. Other contributing factors may include a lack of interest in and/or unfamiliarity with DTC‐GT, a preference to test via a medical professional, fears about potential consequences of results, and matters related to the DTC setting. The latter include rising public concerns about data privacy and security, costs, lack of trust in DTC‐GT companies, and concerns about the validity and accuracy of results.

### 4.2 Factors associated with different types of DTC‐GT

Factors influencing the acceptability, consideration, and intention of DTC‐GT included age, education level, gender, having adopted children or stepchildren, a partner, a chronic disease, or a genetic disease in the family. There was overlap in the factors influencing outcomes for different test types, particularly for age, education level, chronic disease status, and having adopted children/ stepchildren. The most common associated factors were age and education.

Age was associated with at least one outcome for all test types. Older age was associated with lower acceptability, consideration, and intention. Similarly, qualitative responses suggested that older respondents felt less need to test, although some considered DTC‐GT when older i.e., when taking medication (pharmacogenetic testing), or when having a higher disease risk. Our findings are in line with studies in the Dutch (Stewart et al., 2018), Western (Bayer et al., 2021; Cherkas et al., 2010; Mavroidopoulou et al., 2015; Vayena et al., 2014), and non‐Western (Hui et al., 2021) populations. Possible explanations include the greater impact that genetic testing results could have on younger individuals' future lives, and their greater general interest in innovation (Stewart et al., 2018). Younger age has also been associated with reduced apprehension toward testing, including worries regarding personal data (Bloss et al., 2010; Mavroidopoulou et al., 2015).

Further, education was associated with at least one outcome for all test types, apart from sport tests. Higher education was associated with lower acceptability, consideration, and intention to use DTC‐GT without healthcare professional involvement. Respondents with higher education may possess greater genetic knowledge (Morren et al., 2007; Vermeulen et al., 2014), which has been shown to decrease the acceptability of and interest in disease‐related DTC‐GT without a healthcare professional (Stewart et al., 2018). In addition, individuals with higher education have reported increased test‐related reservations (Bloss et al., 2010; Mavroidopoulou et al., 2015). Conversely, greater genetic knowledge has been linked to more positive attitudes toward genetic testing overall (Morren et al., 2007; Vermeulen et al., 2014). Previous studies on the relationship between education and genetic testing are somewhat conflicting. Some studies show that attitudes toward DTC‐GT vary by education level (Hall et al., 2012; Vermeulen et al., 2014), while others show no association (Hui et al., 2021; Stewart et al., 2018).

We observed some areas of discordance between our quantitative and qualitative findings. Qualitative responses provided additional insights into factors influencing test decisions.

Respondents expressed interest in testing for their current or future children's health, consistent with previous studies (Cherkas et al., 2010; Oliveri et al., 2020). However, we found an association only between having adopted children or stepchildren and increased consideration of disease, diet and metabolism, and ancestry tests. A possible explanation could be that respondents recognized that testing has less impact on adopted children and stepchildren due to the lack of shared DNA, possibly making them more willing to test. However, to our knowledge, no studies have been conducted that support this. There was also discordance between qualitative and quantitative responses regarding the influence of family health history on testing decisions. Open‐ended responses indicated family history as a reason to test for one's health, while quantitative results showed no association between having a genetic disease in the family and interest in disease‐related tests. These conflicting results support previous research reporting both positive and negative associations between family disease history and interest in DTC‐GT (Dong et al., 2022; Mählmann et al., 2016; Stewart et al., 2018).

Discordance between qualitative and quantitative results is supported by previous studies (Lowes et al., 2022; Peck et al., 2021) and may arise because open‐ended responses capture individual perceptions, which may not align with those of the broader public. Alternatively, quantitative responses may force respondents to choose a response that may not perfectly represent their opinion.

Another interesting finding was that individuals with chronic diseases were less accepting of health‐related testing (including pharmacogenetics) without the involvement of a healthcare professional but showed increased consideration of taking pharmacogenetic tests in the future. People with a chronic disease may come to the study with different perspectives on the value of health professionals, have pre‐existing trust in healthcare professionals, and are likely taking medications they may wish to optimize. This aligns with open‐ended responses indicating a willingness to test if using medication. The lower acceptability of direct result sharing underscores a preference for involving health professionals in the process, highlighting the perceived need for expert guidance in interpreting results.

### 4.3 Cultural and healthcare context

A crucial factor shaping public perceptions is likely the availability of clinical genetic services in the Netherlands. Genetic counseling is primarily hospital‐based and diagnostic, limiting access to elective genetic tests. This infrastructure may explain the observed preference for professional oversight for health‐related DTC‐GT. In contrast, in countries with limited access to clinical genetics, individuals may more readily turn to DTC‐GT for medical insights, which can drive a different set of perceptions and practices. This distinction helps contextualize the cautious yet curious stance of Dutch respondents and provides a broader framework for understanding the global variability in attitudes toward DTC‐GT.

### 4.4 Implications

Findings from this study suggest that individuals want support with undergoing DTC‐GT, particularly for health‐related tests. Genetic counselors and other healthcare providers trained in genetics are the most suitable professionals to provide support due to their specialized knowledge and the considerable overlap with counseling for traditional genetic testing (Blout Zawatsky et al., 2023). However, given the predominantly diagnostic context in which genetic counseling takes place in the Netherlands, and challenges such as limited time and potential discomfort in addressing DTC‐GT results (Hsieh et al., 2021; Miura et al., 2023), the current feasibility of one‐on‐one counseling for DTC‐GT by genetic counselors is questionable. Genetic counselors' discomfort with DTC‐GT results may stem from a lack of knowledge of or experience with such results, uncertainty about the accuracy of DTC‐GT results, and the belief that discussing DTC‐GT results with consumers is not the best use of clinical time (Hsieh et al., 2021). Despite these challenges, the expertise of genetic counselors can be leveraged in the design of (online) educational materials for DTC‐GT, ensuring that the resources are engaging and accessible to individuals of varying ages and educational backgrounds, including younger and less educated audiences. By tailoring content to accommodate the needs and interests of these groups, such resources can empower potential consumers to make better‐informed decisions, better understand test results, and interpret the implications of the results. Additionally, genetic counselors play a central role in supporting the training of other healthcare professionals, particularly general practitioners, who often serve as the primary contact for individuals seeking advice about DTC‐GT in the Netherlands (Rijksinstituut voor Volksgezondheid en Milieu, 2024). Furthermore, genetic counselors may offer counseling to consumers through DTC‐GT companies or independent telehealth genetic counseling services, and their expertise can support public health campaigns aimed at increasing genetic literacy.

Given that individuals with lower educational levels showed higher acceptability of and interest in DTC‐GT, public health initiatives focused on increasing genetic literacy should be prioritized. These initiatives could involve creating accessible educational materials on DTC‐GT, particularly targeting younger, less educated populations. Strengthening industry standards related to pretest information and informed consent processes is also critical to ensure that individuals, especially those with lower educational backgrounds, are equipped with the knowledge needed to make informed choices.

### 4.5 Strengths and limitations

This study's large sample size and comprehensive analysis offer valuable insights into Dutch attitudes toward DTC‐GT. However, some limitations need addressing. (1) Not all respondents answered the open‐ended questions, which may skew qualitative findings. The broad nature of the qualitative questions might also limit the depth of understanding for specific test types. Further interview or focus group studies would be valuable to better understand the reasons behind the public's DTC‐GT test decisions in the Netherlands. (2) Where possible, the survey on which this study is based used previously validated questionnaires, but also some newly designed items. A small sample was used to successfully pretest the questionnaire. (3) The exclusion of incomplete questionnaires, as well as the provision of participant information materials (including links to DTC‐GT websites), may have positively skewed results. For example, promotional content or claims on DTC‐GT company websites could potentially have skewed responses by shaping respondents' perceptions of the tests or increasing familiarity with the DTC‐GT services. However, these materials were selected to reflect commonly accessible resources that the general public might consult when seeking information about DTC‐GT and were intended to provide a realistic context for participant decision‐making. (4) Different analysis methods were used compared to our previous study (Leerschool et al., 2024), resulting in slightly different significance levels for the association between education and disease‐related test intention. However, the direction and magnitude of the association remained the same. (5) The wording of the acceptability question, which tied the acceptability of testing to the mandatory involvement of a healthcare professional, could have influenced participants' responses. This framing could have been understood as implying that healthcare professional involvement is compulsory, potentially leading participants to perceive DTC‐GT without such involvement as less acceptable. Future studies should consider exploring the acceptability of testing with varying levels of healthcare professional involvement to provide a more nuanced understanding. (6) The study did not differentiate between medically actionable and non‐actionable health conditions in the questionnaire. This distinction is important, as attitudes toward genetic testing may vary depending on how medically actionable a condition is (e.g., limited actionability in the case of Alzheimer's disease). Future studies should aim to specifically examine how the public perceives testing for both types of conditions. (7) The variability in the quality of pre‐ and post‐test materials across DTC‐GT companies and the availability of pre‐ and post‐test genetic counseling support can significantly impact the understanding of test results and the ability to make informed decisions. The study did not assess these variables, but future research should explore how these materials and counseling services influence consumer decisions regarding DTC‐GT.

## 5 CONCLUSION

This study indicates that the Dutch public shows significant interest in health‐related DTC‐GT but generally does not find it acceptable to undergo these tests without support. Our findings show overlap in the factors influencing outcomes for different test types, with lower age and education levels most commonly associated with higher acceptability, consideration and intention of DTC‐GT. Ensuring DTC‐GT information is comprehensible for younger individuals and those with less education is crucial for supporting potential consumers in decision‐making and result interpretation. Genetic counselors could provide valuable expertise in developing these materials.

## AUTHOR CONTRIBUTIONS

Study design was carried out by ARL in collaboration with AW and MZ. The data were collected by the internet research company Flycatcher (Maastricht, The Netherlands) (Flycatcher Internet Research, 2024). All quantitative and qualitative data analyses and the writing of the first draft of the manuscript were carried out by ARL. Qualitative data analyses were carried out with guidance from GG and 10% of codes were checked by AW. AW, GG, and MZ critically revised the manuscript for important intellectual content. All authors approved the final manuscript to be published and agree to be accountable for all aspects of the work in ensuring that questions related to the accuracy or integrity of any part of the work are appropriately investigated and resolved.

## FUNDING INFORMATION

The authors declare that no funds, grants, or other support was received during the preparation of this manuscript.

## CONFLICT OF INTEREST STATEMENT

Anna Roos Leerschool, Anke Wesselius, Gowri Gopalakrishna, and Maurice P. Zeegers declare that they have no conflict of interest.

## ETHICS STATEMENT

Human studies and informed consent: This study protocol was reviewed and approved by the Ethics Review Committee of Maastricht University (FHML‐REC/2021/085). All persons gave online informed consent prior to their inclusion in the study.

Animal studies: No non‐human animal studies were carried out by the authors for this article.

## Supporting information


Table S1

Table S2

Table S3

Table S4

Table S5

Table S6

Table S7

Table S8

Appendix S1

Appendix S2

## Data Availability

The data that support the findings of this study are available from the corresponding author upon reasonable request.
