# Supplementary material for: Acceptability, consideration, intention, and uptake of six common types of direct‐to‐consumer genetic tests in the Netherlands
Source: J Genet Couns. 2025 Nov 25;34(6):e70142. doi: 10.1002/jgc4.70142 (PMC12647929; doi:10.1002/jgc4.70142)
Supplement: Supplementary file 1 — Table S1 [file JGC4-34-0-s003.docx]

**Supplementary Table 1. Acceptability, consideration, intention and actual uptake of six common types of DTC-GT**

| Acceptability | Disease-related  n (%) | Sport  n (%) | Diet and metabolism  n (%) | Ancestry  n (%) | Pharmaco-genetics n (%) | Entertainment n (%) |
| --- | --- | --- | --- | --- | --- | --- |
| Totally unacceptable | 325 (35.8) | 125 (13.8) | 161 (17.8) | 119 (13.1) | 268 (29.5) | 136 (15.0) |
| Somewhat unacceptable | 259 (28.6) | 144 (15.9) | 256 (28.2) | 90 (9.9) | 268 (29.5) | 80 (8.8) |
| Neutral | 148 (16.3) | 283 (31.2) | 221 (24.4) | 239 (26.4) | 164 (18.1) | 239 (26.4) |
| Somewhat acceptable | 101 (11.1) | 214 (23.6) | 179 (19.7) | 250 (27.6) | 123 (13.6) | 205 (22.6) |
| Totally acceptable | 74 (8.2) | 141 (15.5) | 90 (9.9) | 209 (23.0) | 84 (9.3) | 247 (27.2) |
| Consideration |  |  |  |  |  |  |
| Definitely not | 158 (17.4) | 418 (46.1) | 188 (20.7) | 220 (24.3) | 173 (19.1) | 351 (38.7) |
| Probably not | 217 (23.9) | 290 (32.0) | 236 (26.0) | 254 (28.0) | 214 (23.6) | 220 (24.3) |
| Maybe/ maybe not | 265 (29.2) | 122 (13.5) | 266 (29.3) | 242 (26.7) | 277 (30.5) | 185 (20.4) |
| Probably yes | 175 (19.3) | 51 (5.6) | 146 (16.1) | 119 (13.1) | 162 (17.9) | 108 (11.9) |
| Definitely yes | 92 (10.1) | 26 (2.9) | 71 (7.8) | 72 (7.9) | 81 (8.9) | 43 (4.7) |
| Intention |  |  |  |  |  |  |
| Definitely not | 403 (44.4) | 606 (66.8) | 415 (45.8) | 449 (49.5) | 411 (45.3) | 537 (59.2) |
| Probably not | 280 (30.9) | 211 (23.3) | 263 (29.0) | 250 (27.6) | 262 (28.9) | 203 (22.4) |
| Maybe/ maybe not | 168 (18.5) | 72 (7.9) | 173 (19.1) | 154 (17.0) | 170 (18.7) | 112 (12.3) |
| Probably yes | 35 (3.9) | 12 (1.3) | 38 (4.2) | 30 (3.3) | 44 (4.9) | 37 (4.1) |
| Definitely yes | 21 (2.3) | 6 (0.7) | 18 (2.0) | 24 (2.6) | 20 (2.2) | 18 (2.0) |
| Uptake |  |  |  |  |  |  |
| Yes | 8 (0.9) | 3 (0.3) | 8 (0.9) | 22 (2.4) | 3 (0.3) | 6 (0.7) |
