# Supplementary material for: Acceptability, consideration, intention, and uptake of six common types of direct‐to‐consumer genetic tests in the Netherlands
Source: J Genet Couns. 2025 Nov 25;34(6):e70142. doi: 10.1002/jgc4.70142 (PMC12647929; doi:10.1002/jgc4.70142)
Supplement: Supplementary file 2 — Table S2 [file JGC4-34-0-s001.docx]

**Supplementary Table 2** Mean ranks from Friedman tests for acceptability, consideration, and intention of six types of DTC-GT

|  | Mean rank | | |
| --- | --- | --- | --- |
| DTC-GT type | Acceptability | Consideration | Intention |
| Disease-related | 2.57 | 4.10 | 3.75 |
| Pharmacogenetics | 2.85 | 3.99 | 3.77 |
| Diet and metabolism | 3.32 | 3.82 | 3.74 |
| Sport | 3.84 | 2.47 | 2.89 |
| Ancestry | 4.21 | 3.61 | 3.59 |
| Entertainment | 4.21 | 3.01 | 3.28 |
|  | χ²=1089.90, p<0.001 | χ²=874.32, p<0.001 | χ²=476.31, p<0.001 |

Note: Friedman test n=907. Higher mean ranks indicate more favourable ratings.
