# Supplementary material for: Acceptability, consideration, intention, and uptake of six common types of direct‐to‐consumer genetic tests in the Netherlands
Source: J Genet Couns. 2025 Nov 25;34(6):e70142. doi: 10.1002/jgc4.70142 (PMC12647929; doi:10.1002/jgc4.70142)
Supplement: Supplementary file 3 — Table S3 [file JGC4-34-0-s009.docx]

**Supplementary Table 3** Uni- and multivariable analyses for acceptability, consideration and intention of DTC-GT for disease-related purposes

|  |  | **Univariable** | | | **Multivariable** | | |
| --- | --- | --- | --- | --- | --- | --- | --- |
| **Acceptability** |  | **b** | **SE b** | **p-value** | **b** | **SE b** | **p-value** |
| **Gender** | Female | -0.190 | 0.120 | 0.113 | -0.074 | 0.124 | 0.553 |
|  | Male | Ref |  |  | Ref |  |  |
| **Age** | 18-39 | Ref |  |  |  |  |  |
|  | 40-59 | 0.101 | 0.144 | 0.483 |  |  |  |
|  | 60+ | -0.145 | 0.150 | 0.332 |  |  |  |
| **Education** | Low | Ref |  |  | Ref |  |  |
|  | Medium | -0.031 | 0.153 | 0.839 | -0.094 | 0.155 | 0.543 |
|  | High | -0.428 | 0.163 | 0.009 | -0.549 | 0.171 | 0.001 |
| **Having a partner** | Yes | 0.176 | 0.131 | 0.179 | 0.265 | 0.138 | 0.054 |
|  | No | Ref |  |  |  |  |  |
| **Being religious** | Yes | -0.061 | 0.128 | 0.633 |  |  |  |
|  | No | Ref |  |  |  |  |  |
| **Planning to have children** | Yes | -0.184 | 0.157 | 0.241 | -0.064 | 0.167 | 0.700 |
|  | Maybe | 0.510 | 0.280 | 0.069 | 0.608 | 0.288 | 0.035 |
|  | Don’t know | 0.733 | 0.296 | 0.013 | 0.930 | 0.308 | 0.003 |
|  | No | Ref |  |  | Ref |  |  |
| **Having biological children** | Yes | -0.112 | 0.121 | 0.353 |  |  |  |
|  | No | Ref |  |  |  |  |  |
| **Having adopted children or stepchildren** | Yes | 0.166 | 0.188 | 0.378 |  |  |  |
|  | No | Ref |  |  |  |  |  |
| **Genetic disease in the family** | Yes | -0.034 | 0.150 | 0.820 |  |  |  |
|  | I would rather not say/ don’t know | 0.185 | 0.154 | 0.227 |  |  |  |
|  | No | Ref |  |  |  |  |  |
| **Having a chronic disease** | Yes | -0.336 | 0.127 | 0.008 | -0.337 | 0.132 | 0.010 |
|  | I would rather not say/ don’t know | -0.523 | 0.320 | 0.102 | -0.444 | 0.323 | 0.169 |
|  | No | Ref |  |  | Ref |  |  |
| **Self-rated health** | Per 1 point increase in score | 0.035 | 0.069 | 0.606 |  |  |  |
|  |  | **Univariable** | | | **Multivariable** | | |
| **Consideration** |  | **b** | **SE b** | **p-value** | **b** | **SE b** | **p-value** |
| **Gender** | Female | 0.037 | 0.118 | 0.756 |  |  |  |
|  | Male | Ref |  |  |  |  |  |
| **Age in years** | Per 1 year increase | -0.022 | 0.004 | <0.001 | -0.025 | 0.005 | <0.001 |
| **Education** | Low | Ref |  |  | Ref |  |  |
|  | Medium | 0.405 | 0.150 | 0.007 | 0.133 | 0.159 | 0.403 |
|  | High | 0.017 | 0.158 | 0.915 | -0.417 | 0.175 | 0.017 |
| **Having a partner** | Yes | 0.212 | 0.130 | 0.103 | 0.307 | 0.147 | 0.037 |
|  | No | Ref |  |  | Ref |  |  |
| **Being religious** | Yes | -0.206 | 0.127 | 0.100 | -0.119 | 0.130 | 0.362 |
|  | No | Ref |  |  | Ref |  |  |
| **Planning to have children** | Yes | 0.501 | 0.161 | 0.002 | -0.006 | 0.215 | 0.977 |
|  | Maybe | 0.871 | 0.274 | 0.001 | 0.592 | 0.305 | 0.052^a^ |
|  | Don’t know | 0.436 | 0.298 | 0.143 | 0.231 | 0.334 | 0.489 |
|  | No | Ref |  |  | Ref |  |  |
| **Having biological children** | Yes | -0.219 | 0.120 | 0.068 | 0.051 | 0.149 | 0.730 |
|  | No | Ref |  |  | Ref |  |  |
| **Having adopted children or stepchildren** | Yes | 0.340 | 0.188 | 0.070 | 0.486 | 0.197 | 0.013 |
|  | No | Ref |  |  | Ref |  |  |
| **Genetic disease in the family** | Yes | 0.193 | 0.148 | 0.192 | 0.059 | 0.151 | 0.695 |
|  | I would rather not say/ don’t know | 0.189 | 0.152 | 0.215 | 0.146 | 0.157 | 0.352 |
|  | No | Ref |  |  | Ref |  |  |
| **Having a chronic disease** | Yes | -0.024 | 0.126 | 0.846 |  |  |  |
|  | I would rather not say/ don’t know | -0.078 | 0.289 | 0.787 |  |  |  |
|  | No | Ref |  |  |  |  |  |
| **Self-rated health** | Per 1 point increase in score | 0.009 | 0.068 | 0.899 |  |  |  |
|  |  | **Univariable** | | | **Multivariable** | | |
| **Intention** |  | **b** | **SE b** | **p-value** | **b** | **SE b** | **p-value** |
| **Gender** | Female | -0.146 | 0.123 | 0.235 |  |  |  |
|  | Male | Ref |  |  |  |  |  |
| **Age in years** | Per 1 year increase | -0.003 | 0.004 | 0.457 |  |  |  |
| **Education** | Low | Ref |  |  | Ref |  |  |
|  | Medium | -0.060 | 0.155 | 0.697 | -0.071 | 0.158 | 0.653 |
|  | High | -0.321 | 0.165 | 0.052 | -0.341 | 0.175 | 0.052 |
| **Having a partner** | Yes | 0.031 | 0.136 | 0.821 |  |  |  |
|  | No | Ref |  |  |  |  |  |
| **Being religious** | Yes | 0.032 | 0.131 | 0.807 |  |  |  |
|  | No | Ref |  |  |  |  |  |
| **Planning to have children** | Yes | 0.090 | 0.159 | 0.571 | 0.213 | 0.166 | 0.199 |
|  | Maybe | 0.475 | 0.292 | 0.104 | 0.561 | 0.293 | 0.055 |
|  | Don’t know | -0.077 | 0.320 | 0.809 | 0.079 | 0.326 | 0.808 |
|  | No | Ref |  |  | Ref |  |  |
| **Having biological children** | Yes | 0.066 | 0.124 | 0.593 |  |  |  |
|  | No | Ref |  |  |  |  |  |
| **Having adopted children or stepchildren** | Yes | 0.190 | 0.193 | 0.325 |  |  |  |
|  | No | Ref |  |  |  |  |  |
| **Genetic disease in the family** | Yes | 0.151 | 0.153 | 0.326 |  |  |  |
|  | I would rather not say/ don’t know | 0.179 | 0.157 | 0.255 |  |  |  |
|  | No | Ref |  |  |  |  |  |
| **Having a chronic disease** | Yes | -0.010 | 0.131 | 0.938 |  |  |  |
|  | I would rather not say/ don’t know | 0.348 | 0.311 | 0.263 |  |  |  |
|  | No | Ref |  |  |  |  |  |
| **Self-rated health** | Per 1 point increase in score | -0.114 | 0.072 | 0.112 | -0.098 | 0.076 | 0.197 |

Legend: ^a^ Without religion in the model b=0.592, SE b 0.295, p=0.045
