# Supplementary material for: Acceptability, consideration, intention, and uptake of six common types of direct‐to‐consumer genetic tests in the Netherlands
Source: J Genet Couns. 2025 Nov 25;34(6):e70142. doi: 10.1002/jgc4.70142 (PMC12647929; doi:10.1002/jgc4.70142)
Supplement: Supplementary file 4 — Table S4 [file JGC4-34-0-s002.docx]

**Supplementary Table 4** Uni- and multivariable analyses for acceptability, consideration and intention of DTC-GT for sport

|  |  | **Univariable** | | | **Multivariable** | | |
| --- | --- | --- | --- | --- | --- | --- | --- |
| **Acceptability** |  | **b** | **SE b** | **p-value** | **b** | **SE b** | **p-value** |
| **Gender** | Female | 0.222 | 0.118 | 0.061 | 0.072 | 0.129 | 0.574 |
|  | Male | Ref |  |  | Ref |  |  |
| **Age** | 18-39 | Ref |  |  | Ref |  |  |
|  | 40-59 | -0.150 | 0.143 | 0.293 | -0.014 | 0.204 | 0.946 |
|  | 60+ | -0.829 | 0.150 | <0.001 | -0.574 | 0.226 | 0.011 |
| **Education** | Low | Ref |  |  | Ref |  |  |
|  | Medium | 0.370 | 0.151 | 0.014 | 0.195 | 0.160 | 0.222 |
|  | High | 0.455 | 0.159 | 0.004 | 0.192 | 0.178 | 0.280 |
| **Having a partner** | Yes | 0.211 | 0.129 | 0.100 | 0.230 | 0.146 | 0.116 |
|  | No | Ref |  |  | Ref |  |  |
| **Being religious** | Yes | -0.264 | 0.126 | 0.036 | -0.180 | 0.130 | 0.165 |
|  | No | Ref |  |  | Ref |  |  |
| **Planning to have children** | Yes | 0.324 | 0.155 | 0.037 | -0.100 | 0.230 | 0.665 |
|  | Maybe | 0.603 | 0.279 | 0.031 | 0.249 | 0.319 | 0.434 |
|  | Don’t know | 0.575 | 0.312 | 0.065 | 0.237 | 0.356 | 0.506 |
|  | No | Ref |  |  | Ref |  |  |
| **Having biological children** | Yes | -0.239 | 0.120 | 0.047 | -0.041 | 0.148 | 0.782 |
|  | No | Ref |  |  | Ref |  |  |
| **Having adopted children or stepchildren** | Yes | -0.001 | 0.187 | 0.995 |  |  |  |
|  | No | Ref |  |  |  |  |  |
| **Genetic disease in the family** | Yes | 0.337 | 0.149 | 0.023 | 0.353 | 0.157 | 0.024 |
|  | I would rather not say/ don’t know | -0.011 | 0.152 | 0.941 | 0.091 | 0.160 | 0.567 |
|  | No | Ref |  |  | Ref |  |  |
| **Having a chronic disease** | Yes | -0.357 | 0.126 | 0.005 | -0.325 | 0.150 | 0.031 |
|  | I would rather not say/ don’t know | -0.899 | 0.322 | 0.005 | -0.813 | 0.378 | 0.032 |
|  | No | Ref |  |  | Ref |  |  |
| **Self-rated health** | Per 1 point increase in score | 0.205 | 0.069 | 0.003 | 0.051 | 0.083 | 0.539 |
|  |  | **Univariable** | | | **Multivariable** | | |
| **Consideration** |  | **b** | **SE b** | **p-value** | **b** | **SE b** | **p-value** |
| **Gender** | Female | -0.240 | 0.124 | 0.052 | -0.416 | 0.137 | 0.002 |
|  | Male | Ref |  |  | Ref |  |  |
| **Age in years** | Per 1 year increase | -0.021 | 0.004 | <0.001 | -0.017 | 0.006 | 0.002 |
| **Education** | Low | Ref |  |  |  |  |  |
|  | Medium | 0.161 | 0.158 | 0.309 |  |  |  |
|  | High | 0.098 | 0.166 | 0.555 |  |  |  |
| **Having a partner** | Yes | -0.173 | 0.135 | 0.201 |  |  |  |
|  | No | Ref |  |  |  |  |  |
| **Being religious** | Yes | -0.173 | 0.132 | 0.190 | -0.086 | 0.137 | 0.530 |
|  | No | Ref |  |  | Ref |  |  |
| **Planning to have children** | Yes | 0.823 | 0.163 | <0.001 | 0.332 | 0.224 | 0.138^a^ |
|  | Maybe | 0.823 | 0.281 | 0.003 | 0.378 | 0.313 | 0.227 |
|  | Don’t know | 0.242 | 0.326 | 0.457 | -0.173 | 0.359 | 0.629 |
|  | No | Ref |  |  | Ref |  |  |
| **Having biological children** | Yes | -0.357 | 0.126 | 0.004 | 0.001 | 0.152 | 0.997 |
|  | No | Ref |  |  | Ref |  |  |
| **Having adopted children or stepchildren** | Yes | -0.134 | 0.201 | 0.505 |  |  |  |
|  | No | Ref |  |  |  |  |  |
| **Genetic disease in the family** | Yes | -0.011 | 0.154 | 0.944 | 0.014 | 0.166 | 0.935 |
|  | I would rather not say/ don’t know | 0.225 | 0.158 | 0.154 | 0.229 | 0.168 | 0.172 |
|  | No | Ref |  |  | Ref |  |  |
| **Having a chronic disease** | Yes | -0.488 | 0.134 | <0.001 | -0.301 | 0.160 | 0.059 |
|  | I would rather not say/ don’t know | 0.027 | 0.309 | 0.931 | 0.019 | 0.352 | 0.957 |
|  | No | Ref |  |  | Ref |  |  |
| **Self-rated health** | Per 1 point increase in score | 0.210 | 0.072 | 0.003 | 0.076 | 0.084 | 0.368 |
|  |  | **Univariable** | | | **Multivariable** | | |
| **Intention** |  | **b** | **SE b** | **p-value** | **b** | **SE b** | **p-value** |
| **Gender** | Female | -0.255 | 0.139 | 0.067 | -0.418 | 0.150 | 0.005 |
|  | Male | Ref |  |  | Ref |  |  |
| **Age in years** | Per 1 year increase | -0.014 | 0.004 | <0.001 | -0.014 | 0.006 | 0.026 |
| **Education** | Low | Ref |  |  |  |  |  |
|  | Medium | 0.153 | 0.177 | 0.387 |  |  |  |
|  | High | -0.013 | 0.188 | 0.943 |  |  |  |
| **Having a partner** | Yes | -0.132 | 0.151 | 0.382 |  |  |  |
|  | No | Ref |  |  |  |  |  |
| **Being religious** | Yes | -0.026 | 0.148 | 0.861 |  |  |  |
|  | No | Ref |  |  |  |  |  |
| **Planning to have children** | Yes | 0.498 | 0.177 | 0.005 | 0.273 | 0.242 | 0.259 |
|  | Maybe | 0.694 | 0.304 | 0.022 | 0.428 | 0.331 | 0.196 |
|  | Don’t know | 0.151 | 0.358 | 0.673 | -0.113 | 0.389 | 0.771 |
|  | No | Ref |  |  | Ref |  |  |
| **Having biological children** | Yes | -0.199 | 0.140 | 0.156 | 0.101 | 0.170 | 0.550 |
|  | No | Ref |  |  | Ref |  |  |
| **Having adopted children or stepchildren** | Yes | -0.027 | 0.224 | 0.903 |  |  |  |
|  | No | Ref |  |  |  |  |  |
| **Genetic disease in the family** | Yes | 0.143 | 0.172 | 0.406 |  |  |  |
|  | I would rather not say/ don’t know | 0.209 | 0.176 | 0.236 |  |  |  |
|  | No | Ref |  |  |  |  |  |
| **Having a chronic disease** | Yes | -0.339 | 0.152 | 0.025 | -0.201 | 0.172 | 0.244 |
|  | I would rather not say/ don’t know | 0.305 | 0.340 | 0.371 | 0.358 | 0.362 | 0.322 |
|  | No | Ref |  |  | Ref |  |  |
| **Self-rated health** | Per 1 point increase in score | 0.114 | 0.080 | 0.155 | 0.013 | 0.093 | 0.888 |

Legend: ^a^ Without religion in the model b=0.438, SE b=0.221, p=0.047
