# Supplementary material for: Acceptability, consideration, intention, and uptake of six common types of direct‐to‐consumer genetic tests in the Netherlands
Source: J Genet Couns. 2025 Nov 25;34(6):e70142. doi: 10.1002/jgc4.70142 (PMC12647929; doi:10.1002/jgc4.70142)
Supplement: Supplementary file 5 — Table S5 [file JGC4-34-0-s010.docx]

**Supplementary Table 5** Uni- and multivariable analyses for acceptability, consideration and intention of DTC-GT for diet and metabolism

|  |  | **Univariable** | | | **Multivariable** | | |
| --- | --- | --- | --- | --- | --- | --- | --- |
| **Acceptability** |  | **b** | **SE b** | **p-value** | **b** | **SE b** | **p-value** |
| **Gender** | Female | 0.205 | 0.118 | 0.083 | 0.194 | 0.128 | 0.130 |
|  | Male | Ref |  |  | Ref |  |  |
| **Age** | 18-39 | Ref |  |  | Ref |  |  |
|  | 40-59 | -0.102 | 0.142 | 0.471 | -0.022 | 0.203 | 0.915 |
|  | 60+ | -0.533 | 0.148 | <0.001 | -0.354 | 0.224 | 0.113^a^ |
| **Education** | Low | Ref |  |  | Ref |  |  |
|  | Medium | 0.205 | 0.152 | 0.176 | 0.116 | 0.161 | 0.472 |
|  | High | 0.187 | 0.160 | 0.242 | 0.040 | 0.179 | 0.825 |
| **Having a partner** | Yes | 0.135 | 0.129 | 0.296 |  |  |  |
|  | No | Ref |  |  |  |  |  |
| **Being religious** | Yes | -0.281 | 0.127 | 0.027 | -0.215 | 0.130 | 0.097 |
|  | No | Ref |  |  | Ref |  |  |
| **Planning to have children** | Yes | 0.095 | 0.153 | 0.538 | -0.285 | 0.230 | 0.216 |
|  | Maybe | 0.623 | 0.287 | 0.030 | 0.329 | 0.323 | 0.308 |
|  | Don’t know | 0.692 | 0.304 | 0.023 | 0.379 | 0.339 | 0.264 |
|  | No | Ref |  |  | Ref |  |  |
| **Having biological children** | Yes | -0.221 | 0.120 | 0.065 | -0.074 | 0.141 | 0.598 |
|  | No | Ref |  |  | Ref |  |  |
| **Having adopted children or stepchildren** | Yes | 0.042 | 0.188 | 0.823 |  |  |  |
|  | No | Ref |  |  |  |  |  |
| **Genetic disease in the family** | Yes | 0.069 | 0.148 | 0.642 |  |  |  |
|  | I would rather not say/ don’t know | 0.013 | 0.152 | 0.931 |  |  |  |
|  | No | Ref |  |  |  |  |  |
| **Having a chronic disease** | Yes | -0.353 | 0.125 | 0.005 | -0.304 | 0.143 | 0.034 |
|  | I would rather not say/ don’t know | -0.936 | 0.318 | 0.003 | -0.882 | 0.365 | 0.016 |
|  | No | Ref |  |  | Ref |  |  |
| **Self-rated health** | Per 1 point increase in score | 0.127 | 0.069 | 0.063 | 0.015 | 0.082 | 0.858 |
|  |  | **Univariable** | | | **Multivariable** | | |
| **Consideration** |  | **b** | **SE b** | **p-value** | **b** | **SE b** | **p-value** |
| Gender | Female | 0.112 | 0.119 | 0.345 |  |  |  |
|  | Male | Ref |  |  |  |  |  |
| **Age in years** | Per 1 year increase | -0.015 | 0.004 | <0.001 | -0.018 | 0.005 | <0.001 |
| **Education** | Low | Ref |  |  |  |  |  |
|  | Medium | 0.160 | 0.153 | 0.294 |  |  |  |
|  | High | -0.100 | 0.161 | 0.533 |  |  |  |
| **Having a partner** | Yes | 0.067 | 0.131 | 0.611 |  |  |  |
|  | No | Ref |  |  |  |  |  |
| **Being religious** | Yes | -0.179 | 0.127 | 0.158 | -0.084 | 0.129 | 0.517 |
|  | No | Ref |  |  | Ref |  |  |
| **Planning to have children** | Yes | 0.298 | 0.159 | 0.060 | -0.223 | 0.211 | 0.290 |
|  | Maybe | 0.445 | 0.263 | 0.090 | 0.033 | 0.391 | 0.910 |
|  | Don’t know | 0.075 | 0.312 | 0.810 | -0.261 | 0.340 | 0.443 |
|  | No | Ref |  |  | Ref |  |  |
| **Having biological children** | Yes | -0.103 | 0.120 | 0.393 |  |  |  |
|  | No | Ref |  |  |  |  |  |
| **Having adopted children or stepchildren** | Yes | 0.366 | 0.188 | 0.051 | 0.461 | 0.194 | 0.017 |
|  | No | Ref |  |  | Ref |  |  |
| **Genetic disease in the family** | Yes | 0.280 | 0.147 | 0.058 | 0.234 | 0.149 | 0.117 |
|  | I would rather not say/ don’t know | 0.124 | 0.153 | 0.420 | 0.064 | 0.159 | 0.699 |
|  | No | Ref |  |  | Ref |  |  |
| **Having a chronic disease** | Yes | 0.026 | 0.126 | 0.834 |  |  |  |
|  | I would rather not say/ don’t know | -0.218 | 0.310 | 0.482 |  |  |  |
|  | No | Ref |  |  |  |  |  |
| **Self-rated health** | Per 1 point increase in score | -0.053 | 0.069 | 0.443 |  |  |  |
|  |  | **Univariable** | | | **Multivariable** | | |
| **Intention** |  | **b** | **SE b** | **p-value** | **b** | **SE b** | **p-value** |
| **Gender** | Female | -0.029 | 0.123 | 0.811 |  |  |  |
|  | Male | Ref |  |  |  |  |  |
| **Age in years** | Per 1 year increase | 0.004 | 0.004 | 0.326 |  |  |  |
| **Education** | Low | Ref |  |  | Ref |  |  |
|  | Medium | -0.181 | 0.156 | 0.245 | -0.139 | 0.158 | 0.381 |
|  | High | -0.523 | 0.167 | 0.002 | -0.434 | 0.176 | 0.014 |
| **Having a partner** | Yes | 0.121 | 0.137 | 0.379 |  |  |  |
|  | No | Ref |  |  |  |  |  |
| **Being religious** | Yes | 0.072 | 0.132 | 0.585 |  |  |  |
|  | No | Ref |  |  |  |  |  |
| **Planning to have children** | Yes | -0.157 | 0.161 | 0.329 | 0.165 | 0.187 | 0.379 |
|  | Maybe | 0.023 | 0.285 | 0.937 | 0.253 | 0.293 | 0.389 |
|  | Don’t know | -0.598 | 0.344 | 0.082 | -0.223 | 0.358 | 0.553 |
|  | No | Ref |  |  | Ref |  |  |
| **Having biological children** | Yes | 0.299 | 0.125 | 0.017 | 0.262 | 0.145 | 0.070 |
|  | No | Ref |  |  | Ref |  |  |
| **Having adopted children or stepchildren** | Yes | 0.410 | 0.195 | 0.036 | 0.385 | 0.199 | 0.053 |
|  | No | Ref |  |  | Ref |  |  |
| **Genetic disease in the family** | Yes | 0.238 | 0.152 | 0.117 | 0.225 | 0.154 | 0.143 |
|  | I would rather not say/ don’t know | 0.164 | 0.159 | 0.302 | 0.117 | 0.162 | 0.469 |
|  | No | Ref |  |  | Ref |  |  |
| **Having a chronic disease** | Yes | 0.109 | 0.131 | 0.405 |  |  |  |
|  | I would rather not say/ don’t know | 0.394 | 0.329 | 0.231 |  |  |  |
|  | No | Ref |  |  |  |  |  |
| **Self-rated health** | Per 1 point increase in score | -0.196 | 0.073 | 0.007 | -0.131 | 0.077 | 0.090 |

Legend: ^a^ Without religion in the model b=-0.452, SE b=0.221, p=0.041
