# Supplementary material for: Acceptability, consideration, intention, and uptake of six common types of direct‐to‐consumer genetic tests in the Netherlands
Source: J Genet Couns. 2025 Nov 25;34(6):e70142. doi: 10.1002/jgc4.70142 (PMC12647929; doi:10.1002/jgc4.70142)
Supplement: Supplementary file 6 — Table S6 [file JGC4-34-0-s006.docx]

**Supplementary Table 6** Uni- and multivariable analyses for acceptability, consideration and intention of DTC-GT for ancestry

|  |  | **Univariable** | | | **Multivariable** | | |
| --- | --- | --- | --- | --- | --- | --- | --- |
| **Acceptability** |  | **b** | **SE b** | **p-value** | **b** | **SE b** | **p-value** |
| **Gender** | Female | 0.245 | 0.119 | 0.038 | 0.201 | 0.128 | 0.117 |
|  | Male | Ref |  |  | Ref |  |  |
| **Age** | 18-39 | Ref |  |  | Ref |  |  |
|  | 40-59 | 0.113 | 0.143 | 0.432 | 0.229 | 0.154 | 0.137 |
|  | 60+ | -0.455 | 0.148 | 0.002 | -0.182 | 0.171 | 0.287 |
| **Education** | Low | Ref |  |  | Ref |  |  |
|  | Medium | 0.263 | 0.151 | 0.083 | 0.143 | 0.159 | 0.370 |
|  | High | 0.327 | 0.158 | 0.039 | 0.119 | 0.176 | 0.498 |
| **Having a partner** | Yes | 0.260 | 0.130 | 0.045 | 0.234 | 0.136 | 0.086 |
|  | No | Ref |  |  | Ref |  |  |
| **Being religious** | Yes | -0.194 | 0.126 | 0.124 | -0.161 | 0.129 | 0.214 |
|  | No | Ref |  |  | Ref |  |  |
| **Planning to have children** | Yes | -0.053 | 0.157 | 0.735 |  |  |  |
|  | Maybe | 0.147 | 0.280 | 0.600 |  |  |  |
|  | Don’t know | 0.223 | 0.300 | 0.457 |  |  |  |
|  | No | Ref |  |  |  |  |  |
| **Having biological children** | Yes | -0.124 | 0.120 | 0.303 |  |  |  |
|  | No | Ref |  |  |  |  |  |
| **Having adopted children or stepchildren** | Yes | 0.052 | 0.192 | 0.788 |  |  |  |
|  | No | Ref |  |  |  |  |  |
| **Genetic disease in the family** | Yes | 0.270 | 0.148 | 0.067 | 0.267 | 0.156 | 0.086 |
|  | I would rather not say/ don’t know | 0.054 | 0.153 | 0.725 | 0.114 | 0.162 | 0.480 |
|  | No | Ref |  |  | Ref |  |  |
| **Having a chronic disease** | Yes | -0.200 | 0.126 | 0.114 | -0.166 | 0.150 | 0.269 |
|  | I would rather not say/ don’t know | -0.725 | 0.311 | 0.020 | -0.624 | 0.359 | 0.082^a^ |
|  | No | Ref |  |  | Ref |  |  |
| **Self-rated health** | Per 1 point increase in score | 0.184 | 0.069 | 0.008 | 0.118 | 0.082 | 0.154 |
|  |  | **Univariable** | | | **Multivariable** | | |
| **Consideration** |  | **b** | **SE b** | **p-value** | **b** | **SE b** | **p-value** |
| **Gender** | Female | 0.030 | 0.119 | 0.798 |  |  |  |
|  | Male | Ref |  |  |  |  |  |
| **Age in years** | Per 1 year increase | -0.014 | 0.004 | <0.001 | -0.013 | 0.005 | 0.010 |
| **Education** | Low | Ref |  |  |  |  |  |
|  | Medium | 0.120 | 0.152 | 0.432 |  |  |  |
|  | High | -0.096 | 0.158 | 0.541 |  |  |  |
| **Having a partner** | Yes | -0.106 | 0.132 | 0.422 |  |  |  |
|  | No | Ref |  |  |  |  |  |
| **Being religious** | Yes | 0.020 | 0.127 | 0.876 |  |  |  |
|  | No | Ref |  |  |  |  |  |
| **Planning to have children** | Yes | 0.442 | 0.157 | 0.005 | 0.198 | 0.210 | 0.346 |
|  | Maybe | 0.557 | 0.271 | 0.040 | 0.378 | 0.293 | 0.197 |
|  | Don’t know | 0.486 | 0.297 | 0.102 | 0.298 | 0.321 | 0.354 |
|  | No | Ref |  |  | Ref |  |  |
| **Having biological children** | Yes | -0.163 | 0.120 | 0.174 | 0.130 | 0.143 | 0.364 |
|  | No | Ref |  |  | Ref |  |  |
| **Having adopted children or stepchildren** | Yes | 0.250 | 0.190 | 0.187 | 0.419 | 0.193 | 0.030 |
|  | No | Ref |  |  | Ref |  |  |
| **Genetic disease in the family** | Yes | 0.236 | 0.147 | 0.107 | 0.176 | 0.148 | 0.234 |
|  | I would rather not say/ don’t know | 0.469 | 0.154 | 0.002 | 0.457 | 0.155 | 0.003 |
|  | No | Ref |  |  | Ref |  |  |
| **Having a chronic disease** | Yes | -0.020 | 0.126 | 0.871 |  |  |  |
|  | I would rather not say/ don’t know | 0.015 | 0.304 | 0.960 |  |  |  |
|  | No | Ref |  |  |  |  |  |
| **Self-rated health** | Per 1 point increase in score | -0.039 | 0.069 | 0.570 |  |  |  |
|  |  | **Univariable** | | | **Multivariable** | | |
| **Intention** |  | **b** | **SE b** | **p-value** | **b** | **SE b** | **p-value** |
| **Gender** | Female | -0.008 | 0.125 | 0.949 |  |  |  |
|  | Male | Ref |  |  |  |  |  |
| **Age in years** | Per 1 year increase | -0.001 | 0.004 | 0.752 |  |  |  |
| **Education** | Low | Ref |  |  | Ref |  |  |
|  | Medium | -0.239 | 0.158 | 0.130 | -0.255 | 0.161 | 0.112 |
|  | High | -0.417 | 0.166 | 0.012 | -0.426 | 0.177 | 0.016 |
| **Having a partner** | Yes | -0.126 | 0.138 | 0.359 |  |  |  |
|  | No | Ref |  |  |  |  |  |
| **Being religious** | Yes | 0.167 | 0.132 | 0.206 |  |  |  |
|  | No | Ref |  |  |  |  |  |
| **Planning to have children** | Yes | 0.081 | 0.162 | 0.615 | 0.255 | 0.172 | 0.138 |
|  | Maybe | 0.457 | 0.281 | 0.103 | 0.578 | 0.285 | 0.043 |
|  | Don’t know | -0.027 | 0.320 | 0.933 | -0.157 | 0.329 | 0.633 |
|  | No | Ref |  |  | Ref |  |  |
| **Having biological children** | Yes | 0.038 | 0.126 | 0.762 |  |  |  |
|  | No | Ref |  |  |  |  |  |
| **Having adopted children or stepchildren** | Yes | 0.265 | 0.198 | 0.182 | 0.314 | 0.203 | 0.122 |
|  | No | Ref |  |  | Ref |  |  |
| **Genetic disease in the family** | Yes | 0.189 | 0.154 | 0.218 | 0.185 | 0.160 | 0.248 |
|  | I would rather not say/ don’t know | 0.397 | 0.161 | 0.014 | 0.343 | 0.166 | 0.039 |
|  | No | Ref |  |  | Ref |  |  |
| **Having a chronic disease** | Yes | 0.079 | 0.132 | 0.549 | -0.124 | 0.155 | 0.422 |
|  | I would rather not say/ don’t know | 0.490 | 0.327 | 0.130 | 0.211 | 0.340 | 0.535 |
|  | No | Ref |  |  |  |  |  |
| **Self-rated health** | Per 1 point increase in score | -0.172 | 0.073 | 0.018 | -0.160 | 0.086 | 0.061 |

Legend: ^a^ Without religion in the model b=-0.740, SE b=0.329, p=0.025
