# Supplementary material for: Acceptability, consideration, intention, and uptake of six common types of direct‐to‐consumer genetic tests in the Netherlands
Source: J Genet Couns. 2025 Nov 25;34(6):e70142. doi: 10.1002/jgc4.70142 (PMC12647929; doi:10.1002/jgc4.70142)
Supplement: Supplementary file 7 — Table S7 [file JGC4-34-0-s004.docx]

**Supplementary Table 7** Uni- and multivariable analyses for acceptability, consideration and intention of DTC-GT for pharmacogenetics

|  |  | **Univariable** | | | **Multivariable** | | |
| --- | --- | --- | --- | --- | --- | --- | --- |
| **Acceptability** |  | **b** | **SE b** | **p-value** | **b** | **SE b** | **p-value** |
| **Gender** | Female | -0.105 | 0.119 | 0.375 |  |  |  |
|  | Male | Ref |  |  |  |  |  |
| **Age in years** | Per 1 year increase | 0.003 | 0.004 | 0.424 |  |  |  |
| **Education** | Low | Ref |  |  | Ref |  |  |
|  | Medium | -0.127 | 0.151 | 0.401 | -0.144 | 0.153 | 0.345 |
|  | High | -0.386 | 0.160 | 0.016 | -0.420 | 0.166 | 0.011 |
| **Having a partner** | Yes | 0.193 | 0.130 | 0.138 | 0.198 | 0.136 | 0.146 |
|  | No | Ref |  |  | Ref |  |  |
| **Being religious** | Yes | -0.044 | 0.127 | 0.731 |  |  |  |
|  | No | Ref |  |  |  |  |  |
| **Planning to have children** | Yes | -0.340 | 0.157 | 0.030 | -0.291 | 0.165 | 0.078 |
|  | Maybe | 0.242 | 0.284 | 0.394 | 0.302 | 0.294 | 0.303 |
|  | Don’t know | 0.187 | 0.310 | 0.546 | 0.251 | 0.321 | 0.434 |
|  | No | Ref |  |  | Ref |  |  |
| **Having biological children** | Yes | 0.073 | 0.120 | 0.547 |  |  |  |
|  | No | Ref |  |  |  |  |  |
| **Having adopted children or stepchildren** | Yes | 0.237 | 0.191 | 0.215 |  |  |  |
|  | No | Ref |  |  |  |  |  |
| **Genetic disease in the family** | Yes | -0.146 | 0.149 | 0.327 |  |  |  |
|  | I would rather not say/ don’t know | 0.134 | 0.153 | 0.381 |  |  |  |
|  | No | Ref |  |  |  |  |  |
| **Having a chronic disease** | Yes | -0.360 | 0.126 | 0.004 | -0.403 | 0.130 | 0.002 |
|  | I would rather not say/ don’t know | -0.361 | 0.307 | 0.240 | -0.341 | 0.309 | 0.270 |
|  | No | Ref |  |  | Ref |  |  |
| **Self-rated health** | Per 1 point increase in score | 0.026 | 0.068 | 0.708 |  |  |  |
|  |  | **Univariable** | | | **Multivariable**^#^ | | |
| **Consideration** |  | **b** | **SE b** | **p-value** | **b** | **SE b** | **p-value** |
| **Gender** | Female | 0.022 | 0.118 | 0.856 |  |  |  |
|  | Male | Ref |  |  |  |  |  |
| **Age in years** | Per 1 year increase | -0.006 | 0.004 | 0.067 | Definitely not -0.022 | 0.006 | <0.001 |
|  |  |  |  |  | Probably not -0.008 | 0.005 | 0.130 |
|  |  |  |  |  | Maybe/maybe not -0.004 | 0.006 | 0.436 |
|  |  |  |  |  | Probably yes 0.006 | 0.007 | 0.425 |
| **Education** | Low | Ref |  |  | Ref |  |  |
|  | Medium | 0.082 | 0.152 | 0.590 | 0.020 | 0.155 | 0.899 |
|  | High | -0.279 | 0.159 | 0.080 | -0.405 | 0.170 | 0.017 |
| **Having a partner** | Yes | 0.013 | 0.130 | 0.919 |  |  |  |
|  | No | Ref |  |  |  |  |  |
| **Being religious** | Yes | 0.042 | 0.126 | 0.737 |  |  |  |
|  | No | Ref |  |  |  |  |  |
| **Planning to have children** | Yes | 0.084 | 0.156 | 0.593 | -0.034 | 0.209 | 0.870 |
|  | Maybe | 0.570 | 0.269 | 0.034 | 0.542 | 0.292 | 0.072 |
|  | Don’t know | 0.244 | 0.295 | 0.409 | 0.271 | 0.323 | 0.401 |
|  | No | Ref |  |  | Ref |  |  |
| **Having biological children** | Yes | -0.070 | 0.120 | 0.559 |  |  |  |
|  | No | Ref |  |  |  |  |  |
| **Having adopted children or stepchildren** | Yes | 0.101 | 0.191 | 0.595 |  |  |  |
|  | No | Ref |  |  |  |  |  |
| **Genetic disease in the family** | Yes | 0.166 | 0.146 | 0.256 |  |  |  |
|  | I would rather not say/ don’t know | 0.026 | 0.155 | 0.869 |  |  |  |
|  | No | Ref |  |  |  |  |  |
| **Having a chronic disease** | Yes | 0.221 | 0.126 | 0.080 | 0.257 | 0.130 | 0.048 |
|  | I would rather not say/ don’t know | -0.140 | 0.298 | 0.638 | -0.088 | 0.299 | 0.769 |
|  | No | Ref |  |  | Ref |  |  |
| **Self-rated health** | Per 1 point increase in score | -0.080 | 0.069 | 0.244 |  |  |  |
|  |  | **Univariable** | | | **Multivariable** | | |
| **Intention** |  | **b** | **SE b** | **p-value** | **b** | **SE b** | **p-value** |
| **Gender** | Female | -0.099 | 0.123 | 0.421 |  |  |  |
|  | Male | Ref |  |  |  |  |  |
| **Age in years** | Per 1 year increase | 0.009 | 0.004 | 0.014 | 0.009 | 0.006 | 0.123 |
| **Education** | Low | Ref |  |  | Ref |  |  |
|  | Medium | -0.390 | 0.156 | 0.013 | -0.309 | 0.166 | 0.062 |
|  | High | -0.759 | 0.167 | <0.001 | -0.646 | 0.185 | <0.001 |
| **Having a partner** | Yes | -0.037 | 0.136 | 0.785 |  |  |  |
|  | No | Ref |  |  |  |  |  |
| **Being religious** | Yes | 0.176 | 0.132 | 0.183 | 0.084 | 0.136 | 0.535 |
|  | No | Ref |  |  |  |  |  |
| **Planning to have children** | Yes | -0.168 | 0.160 | 0.294 | 0.229 | 0.224 | 0.308 |
|  | Maybe | 0.412 | 0.284 | 0.147 | 0.761 | 0.314 | 0.015 |
|  | Don’t know | -0.356 | 0.323 | 0.271 | 0.078 | 0.359 | 0.828 |
|  | No | Ref |  |  | Ref |  |  |
| **Having biological children** | Yes | 0.193 | 0.125 | 0.122 | 0.076 | 0.151 | 0.616 |
|  | No | Ref |  |  | Ref |  |  |
| **Having adopted children or stepchildren** | Yes | 0.219 | 0.196 | 0.265 |  |  |  |
|  | No | Ref |  |  |  |  |  |
| **Genetic disease in the family** | Yes | 0.221 | 0.152 | 0.145 | 0.228 | 0.161 | 0.155 |
|  | I would rather not say/ don’t know | 0.174 | 0.159 | 0.273 | 0.098 | 0.168 | 0.559 |
|  | No | Ref |  |  | Ref |  |  |
| **Having a chronic disease** | Yes | 0.201 | 0.131 | 0.125 | -0.052 | 0.155 | 0.740 |
|  | I would rather not say/ don’t know | 0.420 | 0.316 | 0.183 | 0.296 | 0.360 | 0.412 |
|  | No | Ref |  |  |  |  |  |
| **Self-rated health** | Per 1 point increase in score | -0.248 | 0.073 | <0.001 | -0.146 | 0.087 | 0.092^a^ |

Legend:

^#^ Partial proportional odds model: For the variable age in years, the reported outcome category (e.g. definitely not) and lower-coded categories are taken as the reference group. Therefore, positive coefficients indicate that higher values of the explanatory variable (age) increase the probability of being in a higher outcome category than the current one. Negative coefficients indicate that older age increases the probability of being in the current or a lower category (Williams 2006).

^a^ Without religion in the model: b=-0.169, SE b=0.086, p=0.050
