# Supplementary material for: Acceptability, consideration, intention, and uptake of six common types of direct‐to‐consumer genetic tests in the Netherlands
Source: J Genet Couns. 2025 Nov 25;34(6):e70142. doi: 10.1002/jgc4.70142 (PMC12647929; doi:10.1002/jgc4.70142)
Supplement: Supplementary file 8 — Table S8 [file JGC4-34-0-s005.docx]

**Supplementary Table 8** Uni- and multivariable analyses for acceptability, consideration and intention of DTC-GT for entertainment

|  |  | **Univariable** | | | **Multivariable** | | |
| --- | --- | --- | --- | --- | --- | --- | --- |
| **Acceptability** |  | **b** | **SE b** | **p-value** | **b** | **SE b** | **p-value** |
| **Gender** | Female | 0.471 | 0.119 | <0.001 | 0.307 | 0.129 | 0.018 |
|  | Male | Ref |  |  | Ref |  |  |
| **Age** | 18-39 | Ref |  |  | Ref |  |  |
|  | 40-59 | -0.156 | 0.143 | 0.277 | -0.154 | 0.205 | 0.453 |
|  | 60+ | -0.922 | 0.150 | <0.001 | -0.716 | 0.227 | 0.002 |
| **Education** | Low | Ref |  |  | Ref |  |  |
|  | Medium | 0.406 | 0.152 | 0.007 | 0.240 | 0.160 | 0.134 |
|  | High | 0.555 | 0.160 | <0.001 | 0.248 | 0.179 | 0.166 |
| **Having a partner** | Yes | 0.073 | 0.130 | 0.573 |  |  |  |
|  | No | Ref |  |  |  |  |  |
| **Being religious** | Yes | -0.329 | 0.126 | 0.009 | -0.186 | 0.130 | 0.152 |
|  | No | Ref |  |  | Ref |  |  |
| **Planning to have children** | Yes | 0.346 | 0.154 | 0.025 | -0.262 | 0.232 | 0.260 |
|  | Maybe | 0.553 | 0.291 | 0.057 | -0.006 | 0.326 | 0.985 |
|  | Don’t know | 0.181 | 0.297 | 0.543 | -0.348 | 0.340 | 0.306 |
|  | No | Ref |  |  | Ref |  |  |
| **Having biological children** | Yes | -0.284 | 0.120 | 0.018 | -0.020 | 0.142 | 0.888 |
|  | No | Ref |  |  | Ref |  |  |
| **Having adopted children or stepchildren** | Yes | 0.001 | 0.188 | 0.997 |  |  |  |
|  | No | Ref |  |  |  |  |  |
| **Genetic disease in the family** | Yes | 0.391 | 0.149 | 0.009 | 0.322 | 0.158 | 0.041 |
|  | I would rather not say/ don’t know | 0.068 | 0.152 | 0.653 | 0.084 | 0.160 | 0.601 |
|  | No | Ref |  |  | Ref |  |  |
| **Having a chronic disease** | Yes | -0.227 | 0.126 | 0.073 | -0.189 | 0.151 | 0.212 |
|  | I would rather not say/ don’t know | -0.523 | 0.324 | 0.106 | -0.457 | 0.381 | 0.229 |
|  | No | Ref |  |  | Ref |  |  |
| **Self-rated health** | Per 1 point increase in score | 0.184 | 0.068 | 0.007 | 0.064 | 0.082 | 0.438 |
|  |  | **Univariable** | | | **Multivariable** | | |
| **Consideration** |  | **b** | **SE b** | **p-value** | **b** | **SE b** | **p-value** |
| **Gender** | Female | 0.021 | 0.120 | 0.864 |  |  |  |
|  | Male | Ref |  |  |  |  |  |
| **Age in years** | Per 1 year increase | -0.010 | 0.004 | 0.007 | -0.003 | 0.005 | 0.553 |
| **Education** | Low | Ref |  |  | Ref |  |  |
|  | Medium | 0.013 | 0.155 | 0.932 | -0.042 | 0.164 | 0.799 |
|  | High | -0.277 | 0.163 | 0.089 | -0.424 | 0.178 | 0.017 |
| **Having a partner** | Yes | -0.012 | 0.133 | 0.926 |  |  |  |
|  | No | Ref |  |  |  |  |  |
| **Being religious** | Yes | -0.209 | 0.129 | 0.104 | -0.190 | 0.133 | 0.152 |
|  | No | Ref |  |  | Ref |  |  |
| **Planning to have children** | Yes | 0.409 | 0.156 | 0.009 | 0.302 | 0.214 | 0.158 |
|  | Maybe | 0.681 | 0.274 | 0.013 | 0.567 | 0.304 | 0.062^a^ |
|  | Don’t know | 0.292 | 0.297 | 0.326 | 0.341 | 0.327 | 0.298 |
|  | No | Ref |  |  | Ref |  |  |
| **Having biological children** | Yes | -0.243 | 0.121 | 0.045 | -0.076 | 0.145 | 0.602 |
|  | No | Ref |  |  | Ref |  |  |
| **Having adopted children or stepchildren** | Yes | 0.096 | 0.196 | 0.624 |  |  |  |
|  | No | Ref |  |  |  |  |  |
| **Genetic disease in the family** | Yes | 0.059 | 0.150 | 0.694 | -0.011 | 0.153 | 0.945 |
|  | I would rather not say/ don’t know | 0.285 | 0.154 | 0.063 | 0.227 | 0.158 | 0.152 |
|  | No | Ref |  |  | Ref |  |  |
| **Having a chronic disease** | Yes | -0.010 | 0.128 | 0.938 |  |  |  |
|  | I would rather not say/ don’t know | 0.213 | 0.320 | 0.505 |  |  |  |
|  | No | Ref |  |  |  |  |  |
| **Self-rated health** | Per 1 point increase in score | 0.043 | 0.069 | 0.535 |  |  |  |
|  |  | **Univariable** | | | **Multivariable** | | |
| **Intention** |  | **b** | **SE b** | **p-value** | **b** | **SE b** | **p-value** |
| **Gender** | Female | -0.083 | 0.130 | 0.524 |  |  |  |
|  | Male | Ref |  |  |  |  |  |
| **Age in years** | Per 1 year increase | 0.001 | 0.004 | 0.761 |  |  |  |
| **Education** | Low | Ref |  |  | Ref |  |  |
|  | Medium | -0.154 | 0.164 | 0.348 | -0.155 | 0.165 | 0.347 |
|  | High | -0.501 | 0.176 | 0.004 | -0.497 | 0.178 | 0.005 |
| **Having a partner** | Yes | -0.051 | 0.143 | 0.723 |  |  |  |
|  | No | Ref |  |  |  |  |  |
| **Being religious** | Yes | 0.062 | 0.139 | 0.654 |  |  |  |
|  | No | Ref |  |  |  |  |  |
| **Planning to have children** | Yes | 0.067 | 0.168 | 0.690 |  |  |  |
|  | Maybe | 0.272 | 0.300 | 0.365 |  |  |  |
|  | Don’t know | 0.366 | 0.352 | 0.298 |  |  |  |
|  | No | Ref |  |  |  |  |  |
| **Having biological children** | Yes | 0.002 | 0.132 | 0.986 |  |  |  |
|  | No | Ref |  |  |  |  |  |
| **Having adopted children or stepchildren** | Yes | 0.172 | 0.203 | 0.397 |  |  |  |
|  | No | Ref |  |  |  |  |  |
| **Genetic disease in the family** | Yes | 0.102 | 0.163 | 0.533 | 0.104 | 0.168 | 0.537 |
|  | I would rather not say/ don’t know | 0.304 | 0.165 | 0.065 | 0.235 | 0.170 | 0.167 |
|  | No | Ref |  |  | Ref |  |  |
| **Having a chronic disease** | Yes | 0.053 | 0.139 | 0.705 | -0.035 | 0.145 | 0.810 |
|  | I would rather not say/ don’t know | 0.594 | 0.342 | 0.082 | 0.520 | 0.348 | 0.135 |
|  | No | Ref |  |  | Ref |  |  |
| **Self-rated health** | Per 1 point increase in score | -0.028 | 0.075 | 0.713 |  |  |  |

Legend: ^a^ Without religion in the model b=0.625, SE b=0.296, p=0.035
