# Supplementary material for: Acceptability, consideration, intention, and uptake of six common types of direct‐to‐consumer genetic tests in the Netherlands
Source: J Genet Couns. 2025 Nov 25;34(6):e70142. doi: 10.1002/jgc4.70142 (PMC12647929; doi:10.1002/jgc4.70142)
Supplement: Supplementary file 10 — Appendix S2 [file JGC4-34-0-s008.docx]

***Supplementary text***

*Introductory text in survey on direct-to-consumer genetic testing*

**What are direct-to-consumer (DTC) genetic tests?**

Nowadays, new DNA tests (genetic tests) are on the market. These tests assess a person’s genetic profile for a variety of information. Companies or research institutes sell these tests directly to a consumer (hence the name direct-to-consumer), without involvement of a health professional. People can order these tests themselves online.

The purposes of testing can be very diverse. In this survey, we focus on six of the most common purposes: (1) disease risk, (2) sports, (3) nutrition and metabolism (4) ancestry (5) response to medication, and (6) fun facts.

**Information on the different types of direct-to-consumer genetic tests**

(1) DTC genetic tests that focus on disease, provide information about your personal risk of developing diseases in the future (such as cancer, heart failure, Alzheimer's disease, type 2 diabetes, etc.). These tests also focus on carrier status of a genetic mutation. A genetic mutation is a "mistake" in the hereditary material (the DNA). A person who is a carrier of the mutation is not sick themselves, but can pass the mutation on to his/her children. See example: <https://www.youtube.com/watch?v=B5D_sP9OlhE>

(2) DTC genetic tests that focus on sports provide information about, for example, your aptitude for a particular sport. For example, based on your DNA, do you have more talent for endurance sports or power sports? See example:

<https://www.youtube.com/watch?v=OYVJbL22i_g>

(3) DTC genetic tests that focus on metabolism and diet predict how you respond to different foods. For example, are you sensitive to caffeine or alcohol? See example:

<https://www.youtube.com/watch?v=LmXb_9J_eEY&list=PLc56t--TrzPawtFnXrzYHAFk_XswJHytK>

(4) DTC genetic testing that focuses on family tree research (genealogy) provide an estimate of your origins and indicate where in the world your ancestors may have come from. Test results include an ethnicity report and identify from which specific groups you descend. These tests can help find new relatives. See example:

<https://www.youtube.com/watch?v=UqC8aStX0ps>

(5) DTC genetic tests that focus on the use of medications, provide information about how your body responds to specific drugs (pharmacogenetics). How quickly people process certain medicines and whether they get side effects varies per person. This is partly due to the parts of your DNA that influence how enzymes function. These tests can help determine which medications, as well as which doses, you respond to best. See example:

<https://www.youtube.com/watch?v=zQ7MRyXweR4>

(6) DTC genetic tests that focus on fun facts are for your own entertainment. These tests mainly provide information about various physical characteristics such as hair/eye colour, but also about your taste/smell preferences and other interesting facts. Examples include: preference for sweet or savoury foods, favourite ice cream flavour, fear of heights, and whether or not you sneeze when looking into bright light. See example:

<https://permalinks.23andme.com/pdf/samplereport_traits.pdf>
